# Transcutaneous spinal cord stimulation neuromodulates pre- and postsynaptic inhibition in the control of spinal spasticity

**DOI:** 10.1016/j.xcrm.2024.101805

**Published:** 2024-11-11

**Authors:** Karen Minassian, Brigitta Freundl, Peter Lackner, Ursula S. Hofstoetter

**Affiliations:** 1 Center for Medical Physics and Biomedical Engineering, Medical University of Vienna, 1090 Vienna, Austria; 2 Neurological Center, Clinic Penzing, Vienna Health Association, 1140 Vienna, Austria; 3 Department of Neurology, Clinic Floridsdorf, Vienna Health Association, 1210 Vienna, Austria

**Keywords:** human, neuroplasticity, non-invasive, low-frequency depression, postsynaptic inhibition, presynaptic inhibition, spasticity, spinal cord circuits, spinal cord injury, spinal cord stimulation

## Abstract

Aside from enabling voluntary control over paralyzed muscles, a key effect of spinal cord stimulation is the alleviation of spasticity. Dysfunction of spinal inhibitory circuits is considered a major cause of spasticity. These circuits are contacted by Ia muscle spindle afferents, which are also the primary targets of transcutaneous lumbar spinal cord stimulation (TSCS). We hypothesize that TSCS controls spasticity by transiently strengthening spinal inhibitory circuit function through their Ia-mediated activation. We show that 30 min of antispasticity TSCS improves activity in post- and presynaptic inhibitory circuits beyond the intervention in ten individuals with traumatic spinal cord injury to normative levels established in 20 neurologically intact individuals. These changes in circuit function correlate with improvements in muscle hypertonia, spasms, and clonus. Our study opens the black box of the carryover effects of antispasticity TSCS and underpins a causal role of deficient post- and presynaptic inhibitory circuits in spinal spasticity.

## Highlights

• Pre- and postsynaptic inhibition are deficient in people with spinal cord injury
• Spinal cord stimulation transiently normalizes spinal inhibitory circuit function
• Spinal cord stimulation concurrently improves clinical measures of spasticity
• Improvements in pre- and postsynaptic inhibition correlate with reduced spasticity

## Graphical abstract

**Figure:**
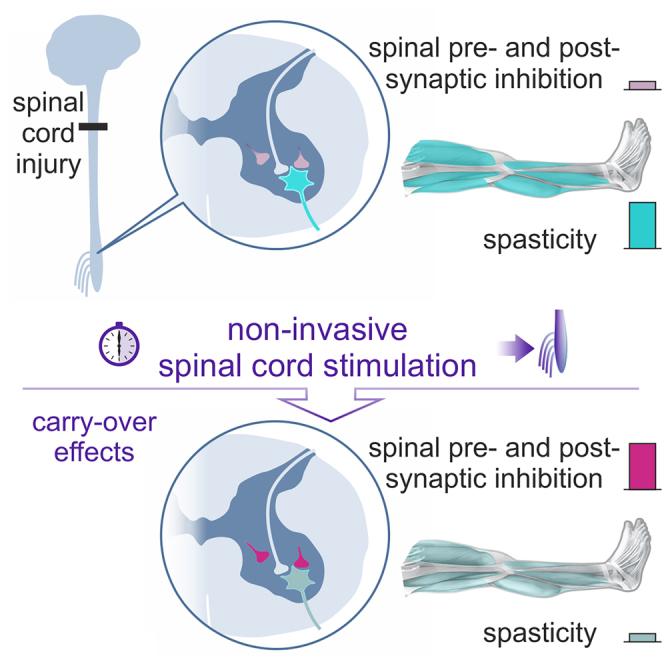

## Teaser

Minassian et al. demonstrate that transcutaneous spinal cord stimulation transiently restores impaired spinal inhibitory function in individuals with spinal cord injury, correlating with improved spasticity. Their findings open the black box of carryover effects of non-invasive neuromodulation and underscore the causal role of deficient pre- and postsynaptic inhibition in spasticity.

## Introduction

Epidural electrical stimulation (EES) has seemingly paradoxical effects on lower-limb motor function following spinal cord injury (SCI), with both enhancing dormant spinal excitability and thereby enabling voluntary control over otherwise paralyzed muscles,^1^^,^^2^ and suppressing the exaggerated excitability that causes spasticity.^3^^,^^4^ These dual effects have also been observed with transcutaneous lumbar spinal cord stimulation (TSCS),^5^ which, similar to lumbar EES, activates large-diameter somatosensory afferents in the posterior roots but non-invasively.^6^^,^^7^ While the motor-enhancing effects have gained considerable attention, research on the impact on spasticity is scarce and has primarily focused on demonstrating clinical efficacy.^8^^,^^9^^,^^10^^,^^11^ Thus far, there has been no exploration into candidate spinal circuits engaged by antispasticity stimulation. Even more elusive are the carryover effects of single sessions of TSCS, which can alleviate spasticity for several hours.^8^^,^^9^^,^^10^

Spasticity affects the majority of individuals after SCI and has a negative impact on many aspects of their lives.^12^^,^^13^ Current clinical management relies primarily on oral medications, despite limited scientific evidence of their efficacy and counterproductive side effects, including muscle weakness, fatigue, and drowsiness.^14^^,^^15^^,^^16^ Spasticity is experienced as abnormal velocity-dependent muscle activation resulting from hyperexcitable stretch reflexes, as well as clonus and muscle spasms (Figure S1).^17^^,^^18^ Identifying the pathophysiological mechanisms underlying spinal spasticity from the cellular to the circuit level in their entirety has remained an ongoing endeavor, with old theories being refuted^19^^,^^20^^,^^21^ and new insights being gained from experimental animal studies.^22^^,^^23^^,^^24^

The common understanding is that spinal spasticity occurs with the profound adaptations in spinal cord circuits caudal to the lesion as a consequence of disrupted descending pathways and deficient monoaminergic modulation of spinal interneurons and motoneurons.^25^^,^^26^ The resulting exaggerated activity in stretch-reflex circuits is considered a core feature of spasticity.^17^^,^^26^ In humans, electrophysiological protocols have been established to selectively explore post- and presynaptic spinal mechanisms that control the excitability of the monosynaptic component of the stretch-reflex circuits.^27^ It was shown that postsynaptic reciprocal Ia inhibition,^28^ presynaptic inhibition,^29^ and low-frequency depression, a measure of rate-dependent depression of neurotransmitter release by Ia afferents,^30^ are all reduced in spastic individuals with chronic SCI. However, no link has been found between these electrophysiological measures of altered circuit function and clinical measures of the severity of spasticity.^31^^,^^32^^,^^33^

**Figure 1:**
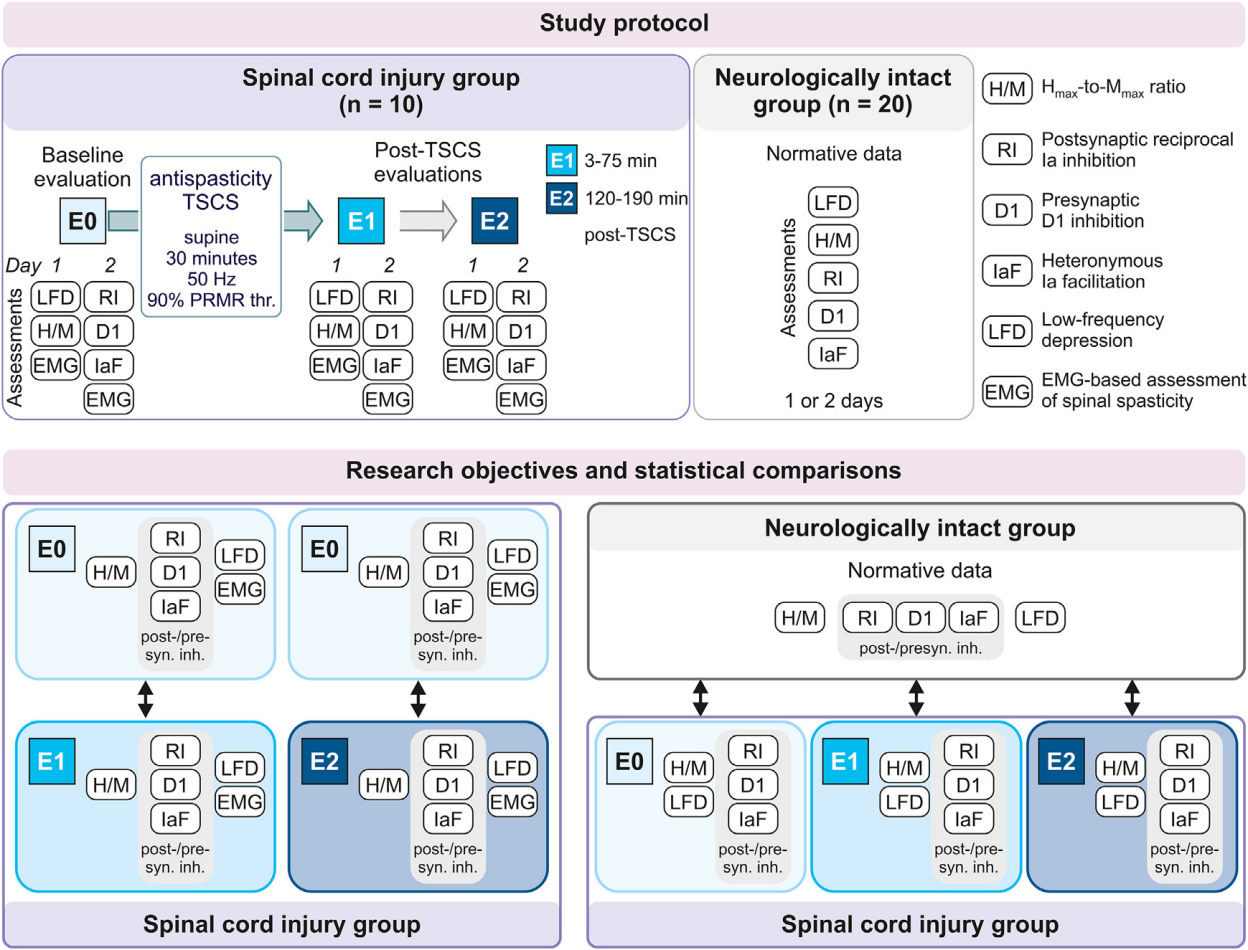
Study protocol The protocol included the electrophysiological assessment of the maximum soleus (SOL)-H reflex (H_max_) to maximum M wave (M_max_) ratio (H/M), postsynaptic reciprocal Ia inhibition (RI), presynaptic D1 inhibition (D1), and heteronymous Ia facilitation (IaF) as well as low-frequency depression (LFD) of the SOL-H reflex in ten individuals with spinal cord injury (SCI). These electrophysiological assessments were supplemented by electromyography (EMG)-based assessments of spinal spasticity. All assessments were performed before (evaluation E0) and twice after (evaluations E1, E2) a 30-min session of antispasticity transcutaneous spinal cord stimulation (TSCS), applied at 50 Hz and at an intensity corresponding to 90% of the posterior root-muscle reflex threshold (PRMR thr.). Data in individuals with SCI were collected on 2 study days. Normative electrophysiological data were collected in 20 neurologically intact individuals. The research objective was to investigate whether antispasticity TSCS would transiently improve the electrophysiological measures of spinal inhibitory function in individuals with SCI compared to baseline. We investigated whether changes in these measures would correlate with changes in the EMG-based measures of spinal spasticity. Additionally, the relationship of the data derived in the SCI group to normative data was studied. Post-/presyn. inh., post- and presynaptic inhibition. See also Table S1 and Figures S1–S3.

The Ia inhibitory interneurons and the trisynaptic spinal circuit underlying post- and presynaptic inhibition, respectively, are immediate transsynaptic targets of Ia muscle spindle afferents.^34^^,^^35^^,^^36^ Ia muscle spindle afferents are also the major neural structures that are electrically activated in the posterior roots by lumbar TSCS and in turn recruit spinal circuits through synaptic transmission.^5^^,^^6^^,^^7^^,^^37^ In the previous studies showing antispasticity effects that outlasted the stimulation for several hours, lumbar TSCS was applied for 30 min at a stimulation frequency of 50 Hz and an amplitude corresponding to 90% of the threshold for eliciting reflex responses in the lower limbs.^8^^,^^9^^,^^10^ We here assumed that these carryover effects of single-session antispasticity TSCS were due to the transient improvement of post- and presynaptic inhibitory circuit function through their repeated Ia afferent-mediated activation,^38^^,^^39^^,^^40^ possibly by temporarily increasing the excitability of the involved interneurons or by potentiating the glutamatergic Ia afferent synapses upon them.^38^ Our research objective was to investigate whether, following antispasticity TSCS, electrophysiological measures of the Ia afferent-mediated motoneuronal excitability would be transiently improved in individuals with SCI compared to baseline (Figure 1). To this end, we assessed the maximum H reflex (H_max_) to maximum M wave (M_max_) ratio, a measure considered to reflect the overall motoneuronal excitability under post- and presynaptic inhibition (Figure S2).^41^^,^^42^ To elucidate the contribution of specific spinal inhibitory mechanisms to the antispasticity effects of TSCS, we investigated whether postsynaptic reciprocal Ia inhibition^43^ and presynaptic inhibition, as assessed by presynaptic D1 inhibition^44^ and heteronymous Ia facilitation,^45^ would be transiently improved after the intervention. We also explored the effects of antispasticity TSCS on a mechanism not mediated by inhibitory circuits, i.e., on low-frequency depression.^46^ We investigated the relationship between the results obtained in individuals with SCI with normative data from neurologically intact individuals.

We applied the same electrophysiological protocols in ten individuals with chronic SCI and spasticity (Table S1) and 20 neurologically intact individuals (Figures 1 and S2). In individuals with SCI, we conducted these protocols before (baseline evaluation E0) and twice after (evaluations E1, 3–75 min, and E2, 120–190 min post TSCS) a 30-min session of TSCS applied at 50 Hz and amplitudes below the threshold for eliciting lower-limb muscle activity (Figure S3). Stimulation parameters were the same as in previous studies of antispasticity TSCS, which had demonstrated carryover effects.^8^^,^^9^^,^^10^ In participants with SCI, the electrophysiological protocols were complemented by electromyography (EMG)-based measures of tonic stretch reflexes, Achilles clonus, and cutaneous-input-evoked spasms (Figure S1). These measures allowed us to correlate changes in the post- and presynaptic spinal inhibitory mechanisms with those in the clinical manifestations of spasticity. Unraveling such interactions would not only open the black box of the carryover effects of antispasticity TSCS but may also contribute to the mechanistic understanding of spinal spasticity per se.

## Results

### TSCS reduced the excitability of the monosynaptic reflex in individuals with SCI, but not below normative levels

We investigated the excitability of soleus (SOL) motoneurons within the monosynaptic reflex arc by assessing the H_max_/M_max_ ratio before and after 30 min of antispasticity TSCS in participants with SCI (Figures 1 and S2). Thereby, H_max_ reflects the excitability of the monosynaptic reflex under post- and presynaptic inhibition and M_max_ is an estimate of the response of the entire motoneuron pool.^41^^,^^42^ TSCS had a large effect on H_max_/M_max_ in evaluation E1, reducing it significantly from E0 to E1 (Table S2). H_max_/M_max_ in E2 did not differ from baseline. There was no statistical difference between H_max_/M_max_ determined in the three evaluations in the SCI group compared with the neurologically intact group. TSCS had thus reduced H_max_/M_max_ in E1, but not below normative values.

### TSCS transiently improved post- and presynaptic inhibition in individuals with SCI to normative levels

**Figure 2:**
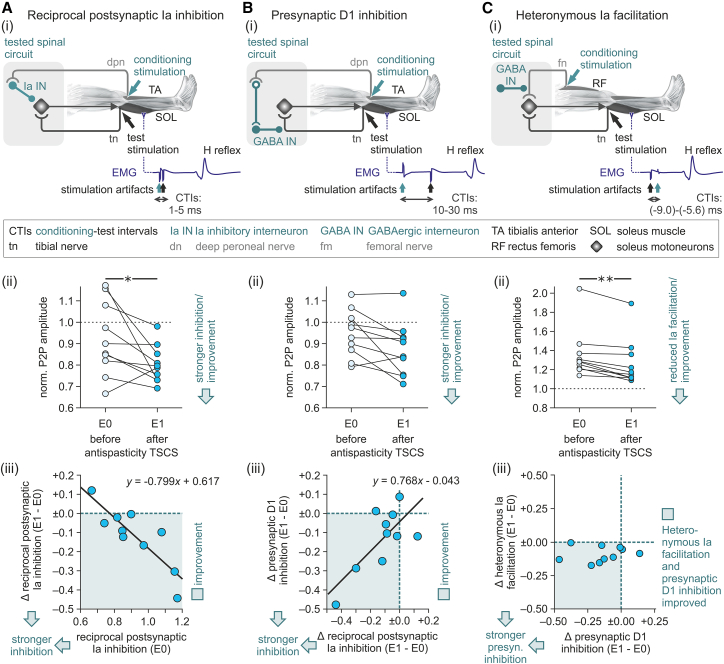
Antispasticity transcutaneous spinal cord stimulation transiently improved post- and presynaptic inhibition in individuals with spinal cord injury (A) (i) Schematic drawing of the disynaptic spinal circuit underlying postsynaptic reciprocal Ia inhibition. For its assessment, the soleus (SOL)-H reflex was elicited by stimulation of the tibial nerve (tn) following a conditioning stimulus applied to the deep branch of the common peroneal nerve (dpn) at conditioning-test intervals (CTIs) of 1–5 ms. (ii) Scatterplots show individual levels of maximum postsynaptic reciprocal Ia inhibition in E0 and E1. Compared to baseline, inhibition was significantly improved in E1. (iii) Maximum baseline inhibition predicted the improvements in E1. In the inserted regression equation, *y* denotes the absolute changes observed in E1 vs. E0 and *x* is the maximum inhibition in E0. (B) (i) Spinal circuit underlying induced presynaptic D1 inhibition. For its assessment, the SOL-H reflex was conditioned by dpn stimulation at CTIs of 10–30 ms. (ii) Individual levels of maximum presynaptic D1 inhibition in E0 and E1. Statistically, presynaptic D1 inhibition did not change in E1 compared to baseline. (iii) Improvements in postsynaptic reciprocal Ia inhibition in E1 predicted improvements in presynaptic D1 inhibition in the same evaluation. In the inserted regression equation, *y* denotes the absolute change in presynaptic D1 inhibition observed in E1 vs. E0 and *x* is the respective change in postsynaptic reciprocal Ia inhibition. (C) (i) Spinal circuit underlying heteronomous Ia facilitation under ongoing presynaptic inhibition, assessed by applying a conditioning stimulation to the femoral nerve (fm) at CTIs of −9.0 to −5.6 ms. (ii) Individual levels of heteronymous Ia facilitation in E0 and E1. Compared to baseline, facilitation was significantly reduced in E1, reflecting increased background presynaptic inhibition. (iii) Relationship between TSCS-induced changes in presynaptic D1 inhibition and heteronymous Ia facilitation in E1 compared to E0. Both measures of presynaptic inhibition were concomitantly improved over baseline in eight of the participants. E0, pre-TSCS evaluation; E1, first post-TSCS evaluation; EMG, electromyographic; TSCS, transcutaneous spinal cord stimulation; ∗*p* < 0.05; ∗∗*p* < 0.001. See also Figures S4 and S6.

We investigated levels of postsynaptic reciprocal Ia inhibition by conditioning the SOL-H reflex with stimulation of the deep branch of the common peroneal nerve at conditioning-test intervals (CTIs) of 1–5 ms (Figure 2A(i)).^43^ Ten conditioned and ten control-H reflexes (without a preceding conditioning stimulus) were collected per CTI. Stimulation amplitudes were set to evoke control-H reflexes with peak-to-peak amplitudes of 20% M_max_.^47^^,^^48^ Maximum postsynaptic reciprocal Ia inhibition was identified as the minimum conditioned-to-control H-reflex size ratio at a CTI of 2 or 3 ms.^43^^,^^49^ Presynaptic inhibition was studied using a dual approach. First, we investigated induced presynaptic D1 inhibition from the deep branch of the common peroneal nerve upon group Ia afferents of SOL at CTIs of 10–30 ms (Figure 2B(i)).^44^ Maximum presynaptic D1 inhibition was identified as the minimum conditioned-to-control H-reflex size ratio at a CTI of 15–25 ms.^29^^,^^44^ Second, we investigated ongoing background presynaptic inhibition based on the amount of heteronymous Ia facilitation from the femoral nerve upon SOL motoneurons at CTIs of −9.0 to −5.6 ms (negative CTIs because the conditioning stimulation site is closer to the spinal cord; Figure 2C(i)).^50^ To obtain sizable, yet uncontaminated monosynaptic facilitation, the CTI selected for assessing heteronymous Ia facilitation was 0.4 ms after the facilitation onset.^45^

Post- and presynaptic inhibition in individuals with SCI were improved following 30 min of antispasticity TSCS (evaluation E1, 3–75 min post TSCS) compared to baseline (evaluation E0, pre-TSCS). Specifically, the factor evaluation (E0, E1) was significant and had a medium effect, while the evaluation × outcome measure interaction was not significant, suggesting that the effect of antispasticity TSCS was consistent across all three outcome measures (Tables 1 and S3).

**Table 1:** Effects of antispasticity transcutaneous spinal cord stimulation on maximum levels of post- and presynaptic inhibition

**SCI group, evaluation E0 vs. E1**	

Factor evaluation (E0, E1)	F_1;54_ = 4.723, *p* = 0.034, $\eta_{p}^{2}$ = 0.080^†††^
Evaluation × outcome measure interaction	F_2;54_ = 0.043, *p* = 0.958, $\eta_{p}^{2}$ = 0.002^†^

**SCI group, evaluation E0 vs. E2**	

Factor evaluation (E0, E2)	F_1;54_ = 0.874, *p* = 0.354, $\eta_{p}^{2}$ = 0.016^††^
Evaluation × outcome measure interaction	F_2;54_ = 0.701, *p* = 0.501, $\eta_{p}^{2}$ = 0.013^††^

**SCI group (E0) vs. neurologically intact group**	

Factor subject group	F_1;84_ = 24.165, *p* < 0.001, $\eta_{p}^{2}$ = 0.223^††††^
Subject group × outcome measure interaction	F_2;84_ = 1.945, *p* = 0.149, $\eta_{p}^{2}$ = 0.044^††^

**SCI group (E1) vs. neurologically intact group**	

Factor subject group	F_1;84_ = 3.727, *p* = 0.057, $\eta_{p}^{2}$ = 0.042^††^
Subject group × outcome measure interaction	F_2;84_ = 2.342, *p* = 0.102, $\eta_{p}^{2}$ = 0.053^††^

**SCI group (E2) vs. neurologically intact group**	

Factor subject group	F_1;84_ = 19.047, *p* < 0.001, $\eta_{p}^{2}$ = 0.185^††††^
Subject group × outcome measure interaction	F_2;84_ = 0.859, *p* = 0.427, $\eta_{p}^{2}$ = 0.020^††^

SCI, spinal cord injury; E0, baseline evaluation before a 30-min session of antispasticity TSCS; E1, E2, post-TSCS evaluations. For statistical comparisons, generalized linear mixed models were run with evaluation and outcome measure as fixed factors for within-SCI group comparisons and with subject group and outcome measure as fixed factors for between-subject group comparisons, respectively; subject was included as random factor in all models. Effect size: ^†^, trivial; ^††^, small; ^†††^, medium; ^††††^, large.

Post hoc Bonferroni-corrected pairwise comparisons showed significantly improved levels of maximum postsynaptic reciprocal Ia inhibition in E1, reflected by lower conditioned-to-control H-reflex size ratios in E1 than E0, *p* = 0.048 (Figure 2A(ii); Table S3). Inhibition was improved in nine of the ten participants with SCI. The exception was participant 10 with the strongest baseline inhibition, who had an SCI classified as grade D on the American Spinal Injury Association Impairment Scale (AIS)^51^ and the highest lower-extremity motor scores. Notably, in three individuals, two of whom had a sensory and motor complete SCI classified as AIS A, reciprocal facilitation rather than inhibition was observed at baseline,^28^ which switched to inhibition in E1. The individuals with absent or the weakest postsynaptic reciprocal Ia inhibition at baseline demonstrated the greatest improvements in E1, as indicated by a linear regression model, F_1;8_ = 23.278, *p* = 0.001, Cohen’s f^2^ = 2.906 (large effect size; Figure 2A(iii)). TSCS additionally improved the time course of postsynaptic reciprocal Ia inhibition over the CTIs of 1–5 ms in E1 compared to E0 (Figure S4A).

Maximum presynaptic D1 inhibition did not show significant changes in E1 compared to baseline following post hoc correction, *p* = 0.160, although it was improved in eight of the ten individuals (Figure 2B(ii)). Yet, TSCS improved the time course of presynaptic D1 inhibition over the CTIs of 5–30 ms in E1 compared to E0 (Figure S4B; Table S3). Improvements in presynaptic D1 inhibition in E1 were strongly positively correlated with improvements in postsynaptic reciprocal Ia inhibition, as indicated by a linear regression model, F_1;8_ = 9.344, *p* = 0.016, Cohen’s f^2^ = 1.169 (large effect size; Figure 2B(iii)).

Heteronymous Ia facilitation was significantly improved in E1 compared to baseline, *p* < 0.001, and in fact, in all ten participants with SCI (Figure 2C(ii); Table S3). Both presynaptic D1 inhibition and heteronymous Ia facilitation were improved over baseline in E1 in eight of the ten participants (Figure 2C(iii)), substantiating that presynaptic inhibition was a basic mechanism targeted by TSCS.^29^^,^^31^

In the second post-TSCS evaluation (E2, conducted 120–190 min post TSCS), statistical analyses demonstrated a small, but not significant effect of the factor evaluation (E0, E2), as well as no significant evaluation × outcome measure interaction (Tables 1 and S3). Postsynaptic reciprocal inhibition and presynaptic inhibition assessed by D1 inhibition and heteronymous Ia facilitation were statistically not different from baseline levels.

**Figure 3:**
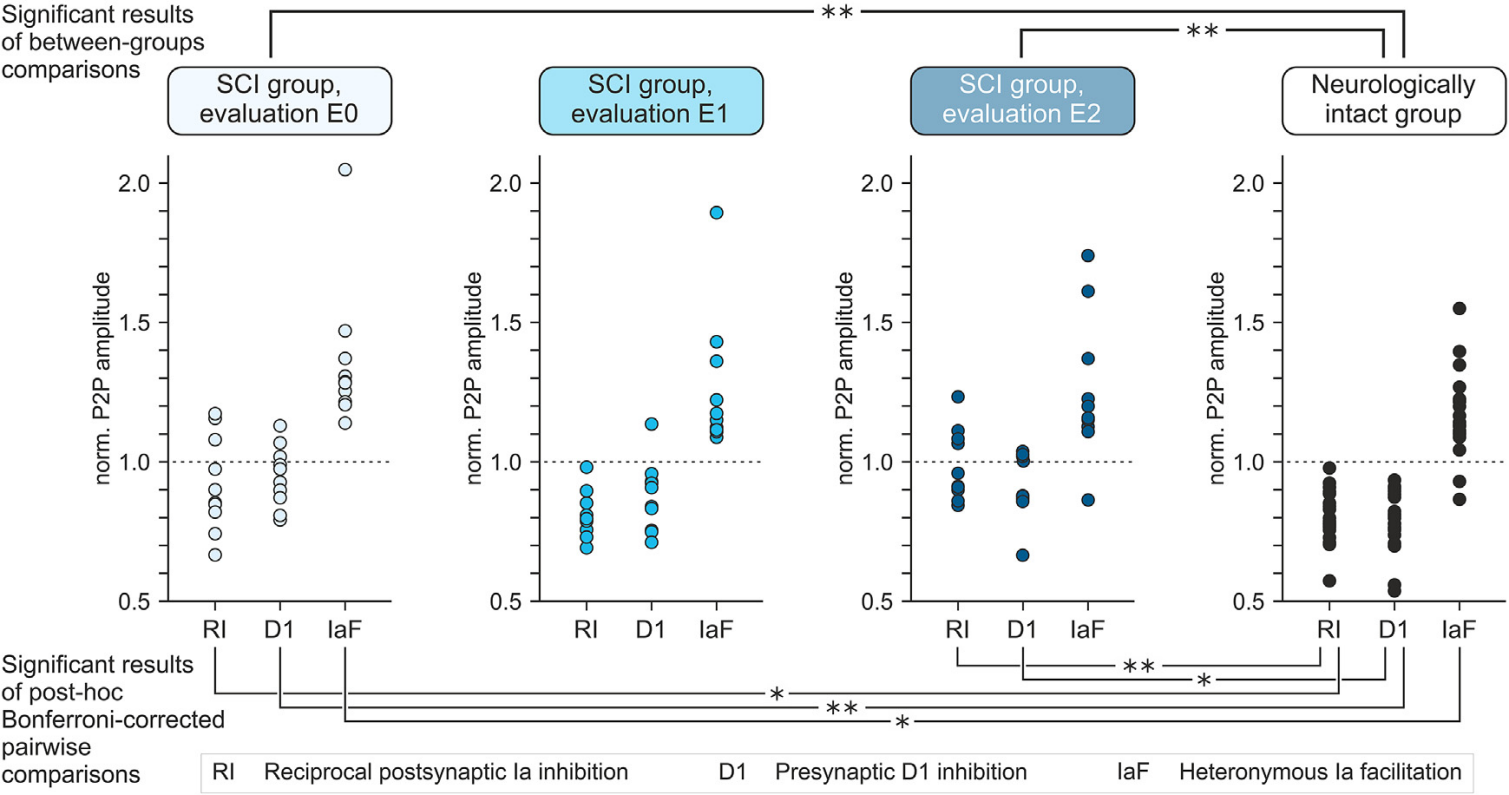
Between-groups comparisons show transient improvement in post- and presynaptic inhibition to normative levels after antispasticity transcutaneous spinal cord stimulation Scatterplots show individual levels of postsynaptic reciprocal Ia inhibition (RI), presynaptic D1 inhibition (D1), and heteronymous Ia facilitation (IaF) of the soleus-H reflex in the spinal cord injury (SCI) group in evaluations E0, E1, and E2 as well as in the neurologically intact group. Post- and presynaptic inhibition were weaker in the SCI than the neurologically intact group in E0 and E2, but not in E1. Post hoc Bonferroni-corrected pairwise comparisons showed lower levels of RI, D1, and IaF in E0 and of RI and D1 in E2 compared to normative levels. E0, pre-TSCS evaluation; E1, E2, post-TSCS evaluations; TSCS, transcutaneous spinal cord stimulation; ∗*p* < 0.05; ∗∗*p* < 0.001. See also Figure S4.

Comparisons between the SCI and neurologically intact groups showed that the baseline levels of post- and presynaptic inhibition were weaker in individuals with SCI (Figures 3 and S4; Table 1). The factor subject group had a large significant effect, while the subject group × outcome measure interaction was not significant, suggesting that SCI had a consistent effect on all three outcome measures (Table 1). Post hoc Bonferroni-corrected pairwise comparisons revealed significant differences for postsynaptic reciprocal Ia inhibition, *p* = 0.020; presynaptic D1 inhibition, *p* < 0.001, and heteronymous Ia facilitation, *p* = 0.025.

In E1, TSCS improved post- and presynaptic inhibition in participants with SCI to levels that did not differ from the neurologically intact group. Neither the factor subject group nor the subject group × outcome measure interaction had significant effects (Tables 1 and S3). Post hoc pairwise comparisons showed no differences between groups for each of the three outcome measures, postsynaptic reciprocal Ia inhibition, *p* = 0.892; presynaptic D1 inhibition, *p* = 0.054; and heteronymous Ia facilitation, *p* = 0.306.

In the second post-TSCS evaluation E2, subject group had a large significant effect on post- and presynaptic inhibition, while the subject group × outcome measure interaction was not significant, suggesting that across outcome measures, levels of inhibition were below those of the neurologically intact group (Tables 1 and S3). Post hoc pairwise comparisons showed significant differences for postsynaptic reciprocal Ia inhibition, *p* = 0.001, and presynaptic D1 inhibition, *p* = 0.004, but not for heteronymous Ia facilitation, *p* = 0.385.

### TSCS did not modulate low-frequency depression

**Figure 4:**
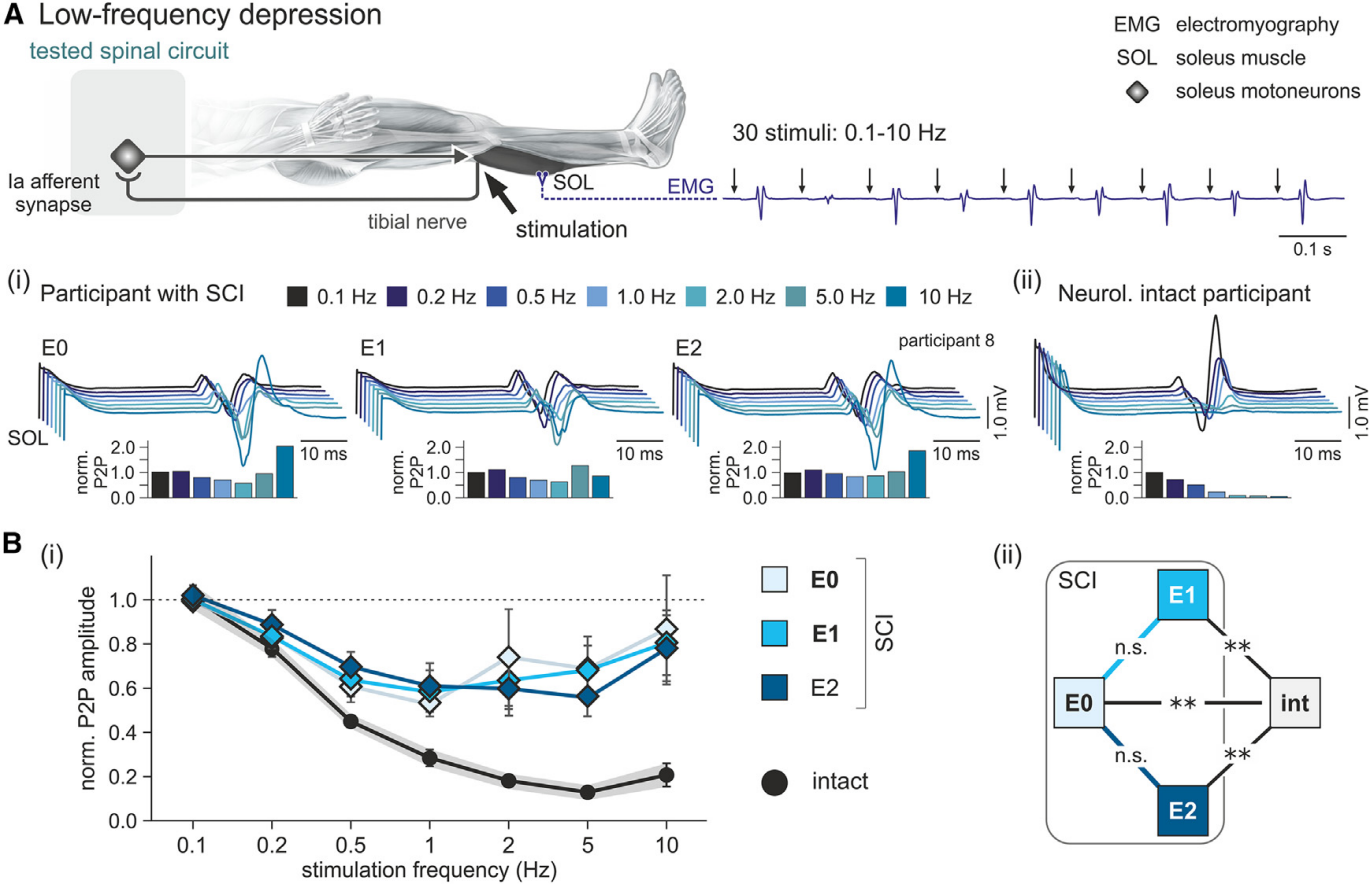
Antispasticity transcutaneous spinal cord stimulation did not modulate low-frequency depression (A) Top: schematic drawing of the repetitively simulated monosynaptic reflex circuit of soleus (SOL). Bottom: exemplary electromyographic recordings of SOL-H reflexes (i) from participant 8 with spinal cord injury (SCI) in evaluations E0, E1, and E2, and (ii) from a neurologically intact participant. Each line is the average of the 11^th^–30^th^ H reflexes elicited at repetition rates as indicated. Insets are mean peak-to-peak (P2P) amplitudes per repetition rate normalized to the H reflexes at 0.1 Hz. (B) (i) Low-frequency depression curves of the H reflex in the SCI group in E0, E1, and E2 (diamonds) and the neurologically intact group (circles). Error bars indicate SE. (ii) Low-frequency depression did not differ between E0 vs. E1 and E0 vs. E2. Low-frequency depression differed significantly between the neurologically intact (int) and the SCI groups in each of the three evaluations. E0, pre-TSCS evaluation; E1, E2, post-TSCS evaluations; n.s., not significant; TSCS, transcutaneous spinal cord stimulation; ∗∗*p* < 0.001.

We investigated low-frequency depression by eliciting SOL-H reflexes with trains of 30 stimuli at frequencies of 0.1–10 Hz and stimulation amplitudes set to evoke control-H reflexes with peak-to-peak amplitudes of 20% M_max_ (Figure 4).^30^^,^^52^ The resulting low-frequency depression curves in E1 and E2, respectively, were not statistically different from E0 (Table S4).

Compared to the neurologically intact group, low-frequency depression curves in all three evaluations in the SCI group were different, with the factor subject group and the subject group × frequency interaction having large effects in all comparisons (Table S4). Hence, low-frequency depression was weaker in the SCI than the neurologically intact group in all three evaluations before and after TSCS.

### Improvements in post- and presynaptic inhibition correlated with improvements in clinical manifestations of spasticity induced by TSCS

**Figure 5:**
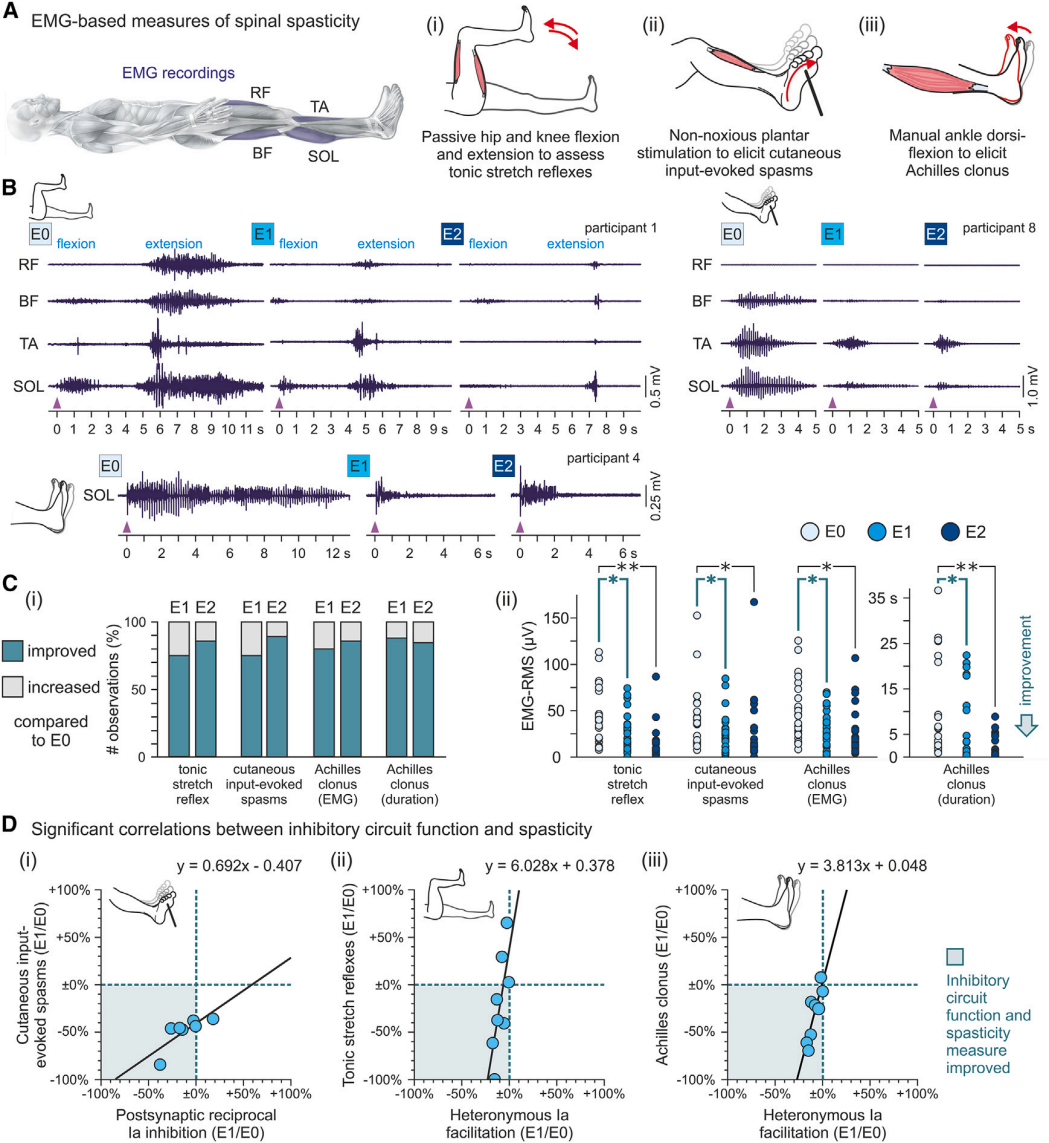
Transcutaneous spinal cord stimulation-induced improvements in spasticity measures correlate with improvements in post- and presynaptic inhibition (A) EMG-based measures of spinal spasticity were acquired from rectus femoris (RF), biceps femoris (BF), tibialis anterior (TA), and soleus (SOL) while (i) tonic stretch reflexes, (ii) cutaneous-input-evoked spasms, and (iii) Achilles clonus were evoked by an examiner. Data were collected twice, on 2 study days. (B) Exemplary recordings show the reduction of the different manifestations of spasticity following TSCS in E1 and E2. Arrowheads indicate onsets of manipulations by the examiner. (C) (i) Turquoise bars illustrate the observation frequency of improvements over baseline for each spasticity measure as indicated, shown separately for E1 and E2, across subjects and study days. (ii) Scatterplots show individual EMG-root-mean-square (RMS) values across muscles of the manipulated lower limb associated with the three spasticity measures as well as Achilles clonus durations. All measures were significantly reduced compared to E0 in both post-TSCS evaluations. Turquoise brackets and asterisks signify significant post hoc Bonferroni-corrected pairwise comparisons between spasticity measures in E0 and E1, black brackets and asterisks between E0 and E2. (D) Scatterplots show significant correlations in E1 between (i) a relative increase in postsynaptic reciprocal Ia inhibition and improvements in cutaneous-input-evoked spasms; a decrease in heteronymous Ia facilitation and (ii) tonic stretch reflexes as well as (iii) Achilles clonus. In the inserted regression equations, *y* denotes the relative change in the EMG-based measure of spasticity as indicated and *x* is the relative change in the respective electrophysiological measure. E0, pre-TSCS evaluation; E1, E2, post-TSCS evaluations; EMG, electromyography; TSCS, transcutaneous spinal cord stimulation; ∗*p* < 0.05; ∗∗*p* < 0.001. See also Figures S5.

TSCS improved EMG-based measures of each of the tested manifestations of spasticity^8^^,^^10^^,^^53^ (Figures 5A and S1), assessed on 2 study days (Figure 1), as exemplified in Figure 5B. Across the 2 study days, TSCS reduced the EMG activity of tonic stretch reflexes tested by passive hip and knee movements in 75.0% of the examinations in evaluation E1 and in 85.7% in evaluation E2 compared to baseline (Figure 5C(i)). Cutaneous-input-evoked spasms were reduced in E1, 75.0%, and E2, 90.0%. Achilles clonus-related EMG activity was reduced in E1, 80.0%, and E2, 85.7%. The duration of Achilles clonus was reduced in E1, 88.0%, and E2, 90.5%, of the examinations. Furthermore, TSCS collectively reduced the EMG-based measures of spasticity in both post-TSCS evaluations compared to baseline (Figure 5C(ii); Tables 2 and S5). For E1, statistical analyses identified a medium significant effect of the factor evaluation, while the evaluation × spasticity measure interaction was not significant (Table 2). Post hoc Bonferroni-corrected pairwise comparisons between E0 and E1 revealed significant differences for all spasticity measures, specifically, for the spasticity-related EMG activity of tonic stretch reflexes, *p* = 0.026; cutaneous-input-evoked spasms, *p* = 0.041; and Achilles clonus, *p* = 0.029; as well as for the duration of Achilles clonus, *p* = 0.001. Similarly, for E2, evaluation had a medium significant effect, while the evaluation × spasticity measure interaction was not significant (Table 2). Post hoc Bonferroni-corrected pairwise comparisons between E0 and E2 revealed significant differences for all spasticity measures, specifically, for the spasticity-related EMG activity tonic stretch reflexes, *p* < 0.001; cutaneous-input-evoked spasms, *p* = 0.001; and Achilles clonus, *p* = 0.031; as well as for the duration of Achilles clonus, *p* < 0.001.

**Table 2:** Effects of antispasticity transcutaneous spinal cord stimulation on electromyography-based measures of clinical manifestations of spasticity

**SCI group, evaluation E0 vs. E1**	

Factor evaluation (E0, E1)	F_1;193_ = 16.760, *p* < 0.001, $\eta_{p}^{2}$ = 0.080^†††^
Evaluation × spasticity measure interaction	F_3;193_ = 1.243, *p* = 0.295, $\eta_{p}^{2}$ = 0.019^††^

**SCI group, evaluation E0 vs. E2**	

Factor evaluation (E0, E2)	F_1;183_ = 20.297, *p* < 0.001, $\eta_{p}^{2}$ = 0.100^†††^
Evaluation × spasticity measure interaction	F_3;179_ = 1.486, *p* = 0.220, $\eta_{p}^{2}$ = 0.024^††^

EMG, electromyography; RMS, root mean square; SCI, spinal cord injury; evaluation E0, baseline evaluation before a 30-min session of antispasticity TSCS; E1, E2, post-TSCS evaluations. For comparisons, generalized linear mixed models with evaluation and spasticity measure as fixed factors and subject as random factor were run. Effect size: ^††^, small; ^†††^, medium.

We next investigated whether these improvements in the EMG-based measures of spasticity following TSCS would correlate with the increased levels of post- and presynaptic inhibition (Figures 5D and S5). Indeed, following TSCS in evaluation E1, the increase in postsynaptic reciprocal Ia inhibition correlated strongly with the reduction in cutaneous-input-evoked spasms, r = 0.782, *p* = 0.038 (Figure 5D(i)). In addition, the reduction in heteronymous Ia facilitation correlated strongly with the reduction in tonic stretch reflexes, r = 0.710, *p* = 0.049 (Figure 5D(ii)), as well as Achilles clonus-related EMG activity, r = 0.866, *p* = 0.005 (Figure 5D(iii)).

## Discussion

Our electrophysiological investigation unveiled deficiencies in post- and presynaptic inhibitory mechanisms among the participants with chronic SCI and spasticity, consistent with previous observations in this subject population.^28^^,^^29^ TSCS engaged the underlying inhibitory circuits to transiently improve their diminished function. In parallel, TSCS improved EMG-based measures of tonic stretch reflexes, cutaneous-input-evoked spasms, and Achilles clonus. These improvements in spasticity were correlated with the increased levels of post- and presynaptic inhibition.

The H_max_/M_max_ ratio is regarded a measure of the Ia afferent-mediated motoneuronal excitability of SOL, which depends on the degree of post- and presynaptic inhibition.^41^^,^^42^ Counterintuitively, we found that H_max_/M_max_ at baseline was not larger in the SCI than the neurologically intact group. Similar findings have been previously reported in individuals with chronic traumatic SCI.^31^^,^^54^^,^^55^ H_max_, which is evoked at submaximal electrical stimulation in humans, is mainly due to the activation of slow-twitch motor units.^56^ Chronic paralysis results in a slow-to-fast twitch fiber type conversion in the SOL,^57^ which could decrease the H_max_/M_max_ ratio^56^ and thus mask a physiologically increased monosynaptic reflex excitability. Here, TSCS still transiently reduced H_max_/M_max_ in the SCI group, indicative of improved post- and presynaptic inhibition, but not below normative levels of the neurologically intact group.

We investigated postsynaptic inhibition as the short-latency reciprocal Ia inhibition of the SOL-H reflex induced by conditioning stimulation of the deep branch of the common peroneal nerve.^43^ Previous studies have shown that the inhibition is maximal at CTIs of 2 or 3 ms, reducing the H reflex by an average of 11.1%–14.9% in neurologically intact individuals.^43^^,^^49^^,^^58^^,^^59^ Various degrees of postsynaptic reciprocal Ia inhibition have been documented in the literature among spastic individuals with SCI, which may imply a dependence on the residual function in chronic SCI.^59^ Maximum inhibition has been reported to be greater than in neurologically intact individuals (H reflex reduced by 32.6%; five individuals with ambulatory SCI),^54^ to be weaker (6%; five AIS C, five AIS D),^49^ or even replaced by facilitation (in seven of 11 participants with a complete SCI).^28^ Our baseline results fit in well with these findings. The strongest inhibition (by 33.5%) was seen in the only participant with a sensory and motor incomplete SCI classified as AIS D, whereas two of the three participants who responded with reciprocal facilitation had a complete SCI classified as AIS A.

We investigated presynaptic inhibition using two protocols, the D1 method to measure induced presynaptic inhibition^44^ and the method of heteronymous Ia facilitation to measure ongoing background presynaptic inhibition.^45^ Stimulation of flexor Ia afferents of the deep peroneal nerve produces presynaptic inhibition of the SOL-H reflex circuit.^44^ The resulting presynaptic D1 inhibition peaks at ∼20 ms, with maximum depression reported to amount to 69.6% ± 15.4%^29^ or 79.1% ± 13.5%^60^ of the (unconditioned) control-H reflex size in neurologically intact individuals. The same studies showed that presynaptic D1 inhibition was significantly weaker in spastic individuals with incomplete SCI (81.2% ± 7.8%; five AIS C, 15 AIS D)^29^ or with various types of spinal cord lesions or diseases (91.7% ± 9.5%).^60^ The baseline level of presynaptic D1 inhibition in our SCI cohort of 90.7% ± 6.3% is consistent with these findings, more closely matching those of Kagamihara and Masakado^60^ who used experimental protocols comparable to the present study. Stimulation of the femoral nerve produces heteronymous Ia facilitation of the SOL-motoneuron pool. The effect is monosynaptic for the first 0.5 ms, during which the increase in H-reflex size can be used to estimate the level of background presynaptic inhibition of the Ia axon terminals from the femoral nerve.^45^ Greater H-reflex facilitation reflects weaker presynaptic inhibition. Facilitation to 111.0% ± 7.1% of the control-H reflex was reported in neurologically intact individuals, while facilitation was greater in individuals with incomplete SCI, amounting to 119.2% ± 9.3%.^29^ The baseline level of heteronymous Ia facilitation in our SCI cohort was 135.7% ± 8.2%. Perhaps the control-H reflex sizes used (50% H_max_^29^ vs. 20% M_max_ in the present study) had influenced the sensitivity of the reflexes to facilitation.^48^ Another earlier study had shown significantly greater heteronymous Ia facilitation in a group of individuals with SCI compared to controls.^31^ Levels of heteronymous Ia facilitation, given as percentage of M_max_, were lower than those in the present study and could be inter alia related to different post-SCI durations of the respective study participants (median of 5 months^31^ versus 6.5 years in the present study).

We applied antispasticity TSCS for 30 min at 50 Hz and an amplitude corresponding to 90% of the threshold for eliciting posterior root-muscle (PRM) reflexes^5^^,^^61^ in the lower-limb muscles. These parameters were originally motivated by early studies of electrical stimulation of proprioceptive afferents. When applied to peripheral nerves at such frequency and duration, stimulation was found to induce carryover effects in sensorimotor circuits lasting for up to 2 h.^62^^,^^63^^,^^64^ Later studies of TSCS using the same parameters found antispasticity effects that also persisted for several hours after application.^8^^,^^9^^,^^10^^,^^61^ Here, 50-Hz TSCS transiently increased postsynaptic reciprocal Ia inhibition and reduced heteronymous Ia facilitation compared to baseline in individuals with SCI and spasticity. Compared to the neurologically intact individuals, postsynaptic reciprocal Ia inhibition and presynaptic D1 inhibition in the SCI group were improved to levels that did not differ from normative values for a median duration of 75 min and heteronymous Ia facilitation for a median duration of 190 min. The concomitant increase in presynaptic D1 inhibition and decrease in heteronymous Ia facilitation substantiated that presynaptic inhibition was a basic mechanism targeted by TSCS and ruled out changes in the recruitment gain of SOL motoneurons as an alternative explanation.^29^^,^^31^ The observed carryover effects of TSCS indicate induced plasticity within the spinal inhibitory circuits. In the following paragraphs, we will discuss that TSCS activates excitatory group Ia fibers immediately afferent to interneurons within the inhibitory circuits. Their activation at 50 Hz and for 30 min would lead to a repeated pairing of presynaptic stimuli with postsynaptic depolarization. This timing of pre- and postsynaptic activity could strengthen the synapses between the Ia afferents and their target interneurons within the spinal inhibitory circuits, according to a long-term potentiation-like phenomenon.^40^ In parallel, the repeated activity of the interneurons could increase their excitability.^38^

Group Ia muscle spindle afferents in the posterior roots/rootlets are the main targets of TSCS.^5^^,^^6^^,^^7^ A fraction of the electric current induced by each TSCS pulse traverses the spinal column, largely through the ligaments, cerebrospinal fluid, and intervertebral discs.^37^^,^^65^ Relatively high current densities develop in the cerebrospinal fluid, in which the roots are immersed. The orientation of the posterior rootlets within the electric field, together with local inhomogeneities in electrical conductivity, creates stimulation hotspots of primary afferents at their entries into the spinal cord, with group Ia afferents having the lowest thresholds.^6^^,^^66^

Group Ia afferents have direct projections to alpha-motoneurons and several types of spinal interneurons. In cats, it was shown that they produce a particularly strong activation of the Ia inhibitory interneurons within the disynaptic reciprocal inhibitory circuit.^34^ In humans, the activation of postsynaptic reciprocal Ia inhibition by electrical stimulation of group Ia fibers has low thresholds and can be induced even with stimuli subthreshold for eliciting an H reflex.^35^^,^^67^ The transient increase in postsynaptic reciprocal Ia inhibition by TSCS could therefore have been caused by the stimulation of the group Ia fibers in the posterior rootlets and potentiation of their glutamatergic synapses on the Ia inhibitory interneurons. Increased excitability of the Ia interneurons by their repeated activation or potentiation of their inhibitory synapses on motoneurons could have also been involved.^38^

Another immediate target of group Ia afferents and hence a possible site of potentiation is the trisynaptic spinal pathway underlying presynaptic inhibition, with a first-order glutamatergic interneuron and a last-order GABAergic interneuron with axo-axonic synapses.^36^ The classical theory of presynaptic inhibition, largely established in rat and cat experiments, is the depolarization of intraspinal Ia fiber endings by GABA_A_ receptor activation (primary afferent depolarization, PAD).^68^ The increased membrane conductance reduces the amplitude of action potentials entering the Ia afferent terminals, resulting in reduced neurotransmitter release.^68^ A recent rodent study suggests that GABA_A_ receptors are rather activated at nodes of Ranvier to facilitate action potential propagation through intraspinal branchpoints of Ia afferent projections.^69^ The nodal PADs may trigger action potentials by themselves, and the orthodromic action potentials conducted toward the terminals may reduce subsequent neurotransmitter release^70^ via GABA_B_ receptor-mediated inhibitory processes^71^ or longer-lasting mechanisms of post-activation depression.^46^

In accordance with previous literature, low-frequency depression, i.e., the rate-dependent depression of trains of H reflexes evoked with increasing frequencies between 0.1 and 10 Hz,^52^ was weaker in the SCI group than in the neurologically intact group.^30^^,^^32^^,^^72^ Low-frequency depression was not improved following TSCS. The mechanism underlying low-frequency depression is presynaptic in origin, lasting up to 10 s, and restricted to the same Ia afferents as excited by the conditioning stimuli,^46^ hence termed homosynaptic depression or post-activation depression.^73^^,^^74^ The classical theory is that repeated activation of the same synapses at increasing stimulation frequencies reduces the probability of quantal neurotransmitter release.^75^ To test low-frequency depression, we stimulated afferents from SOL peripherally in the tibial nerve, while TSCS targeted them within the L5/S1 roots.^76^ The 50-Hz TSCS was applied with an intensity subthreshold to evoke PRM reflexes^5^^,^^61^ in the lower-limb muscles (cf. Figure S3). For this reason, it most likely recruited only a fraction of the same Ia afferents that were activated by the suprathreshold stimulation of the tibial nerve when assessing low-frequency depression of SOL-H reflexes. Such discrepancy would have left a large proportion of the afferent synapses on the SOL-motoneurons involved in homosynaptic depression unconditioned by TSCS. An alternative explanation could be that the carryover effects of TSCS largely involve long-term potentiation-like processes affecting Ia afferent synapses on interneurons within the post- and presynaptic inhibitory circuits, rather than adaptations of neurotransmitter release by the afferents as a response to their repeated activation.

While previous studies have linked various spinal inhibitory mechanisms to spasticity just on the basis of their deficiency in chronic SCI, no convincing relationship has been found between individual electrophysiological measures of altered circuit function and the severity of spasticity.^33^ Studies found that neither reduced presynaptic inhibition^31^ nor post-activation depression^32^ correlated with the severity of spasticity measured by the Modified Ashworth Scale (MAS)^77^ in individuals with SCI, a standard clinical scale used to rate muscle hypertonia. A possible interpretation is that the chronic state of muscle hypertonia cannot be adequately explained by individual electrophysiological measures alone. The acute improvements in spasticity observed here, however, did correlate with the acute improvements in inhibitory circuit function following TSCS.

The transient increase in postsynaptic reciprocal Ia inhibition correlated with the reduction in cutaneous-input-evoked muscle spasms.^20^^,^^78^ Muscle spasms in both humans and experimental animals have been associated with enhanced activation of intrinsic persistent inward currents (PICs) in motoneurons, which generate prolonged depolarizations (plateau potentials) leading to self-sustained firing.^79^^,^^80^ PIC activity can be terminated by hyperpolarization of the motoneuronal membrane potential through an increase in postsynaptic inhibition, such as postsynaptic reciprocal Ia inhibition.^81^^,^^82^^,^^83^

The reduction in heteronymous Ia facilitation following TSCS correlated with a reduction in tonic stretch reflexes and Achilles clonus. Tonic stretch reflexes and Achilles clonus are both initiated and maintained within the stretch reflex circuit, and aberrant background facilitation of motoneurons is likely to contribute to its pathologically increased excitability.^84^^,^^85^

The electrophysiological methods of this study were selected with the presumption that the major TSCS-induced effects could be explained by the Ia afferent-mediated synaptic activation of interneurons within post- and presynaptic inhibitory circuits. Our discussion of potential mechanisms was accordingly focused on long-term potentiation-like processes and modulation of the excitability of the engaged interneurons. Yet, this is not to exclude other possible explanations. The observation of reciprocal facilitation instead of inhibition in three of our participants with SCI^28^ could be explained by a chronic downregulation of the potassium-chloride cotransporter KCC2 of motoneurons, a resulting disruption of the Cl^−^ homeostasis, and a switch to glycine receptor-mediated depolarization instead of hyperpolarization.^23^ The reversal from facilitation to inhibition in these participants following TSCS would then mean that mechanisms were activated that could acutely restore Cl^−^ homeostasis. Animal studies have very recently begun to directly address the spinal pathways and molecular mechanisms activated by TSCS.^86^^,^^87^

### Limitations of the study

This study was not blinded or sham controlled. Providing an appropriate sham condition in clinical trials involving a medical device is inherently challenging. A prominent example is conventional tonic EES for the treatment of chronic pain.^88^ Such tonic stimulation elicits paresthesias that have been directly associated with an effective treatment. These sensory cues have impeded the conduct of sham-controlled or blinded studies.^89^^,^^90^ Similarly, tonic TSCS produces characteristic paresthesias in the lower-limb dermatomes as well as neuromuscular stimulation of the trunk throughout the duration of its application. Sham conditions such as those employed in studies of sensation-free direct-current brain or spinal cord stimulation^91^ are therefore inapplicable. While blinding is particularly important for subjective outcome measures, its necessity is diminished when assessing objective measures collected without the presence of an assessor,^92^ such as the electrophysiological measures in this study. Potential assessor bias cannot be completely excluded in the EMG-based assessment of spasticity. However, as opposed to clinical ratings of spasticity, the outcome measures here were not determined by the assessor but were objectively calculated after data collection had been completed.

The focus of the present study was the electrophysiological investigation of spinal inhibitory mechanisms that may be involved in the antispasticity effects of TSCS, complemented by EMG-based assessments of spasticity. The standardized clinical evaluation of muscle hypertonia based on the MAS,^77^ previously shown to be improved by antispasticity TSCS,^10^^,^^53^ was only determined at the time of enrollment of the participants with SCI, but not used as an outcome measure.

The sequence of electrophysiological and EMG-based assessments was kept constant across the three evaluations performed in the SCI group, and a potential influence of the order of testing and time elapsed after TSCS on the outcome measures could be considered in future studies.

There was no prior data in the literature to allow prospective sample size calculation. To assess the power of our study, we conducted a retrospective sample size calculation using GLIMMPSE,^93^ based on the observed effects of TSCS on pre- and postsynaptic inhibition. We found that our sample size of 10 resulted in 84.3% statistical power with an α-level of 0.05 to detect an effect of TSCS on pre- and postsynaptic inhibition in a generalized linear mixed model with evaluation (E0, E1) and outcome measure as fixed factors. However, considering the intrinsic heterogeneity of the population of individuals living with SCI, confirmatory studies with larger sample sizes will be crucial to improve the precision of parameter estimates.

Several factors could have influenced the levels of spinal inhibition as well as the antispasticity effects of TSCS in our SCI group, including antispasticity medication, SCI severity, and age (Figure S6). Current clinical studies in SCI do not necessarily exclude participants who are on antispasticity medication, provided that they have taken their last dose approximately 12 h before participation^29^ or have maintained stable medication for several weeks.^9^ Four of the individuals with SCI in the present study had taken antispasticity medication 12–24 h prior to their participation (cf. STAR Methods and Table S1). There was no clear separation between levels of spinal inhibition in individuals with or without a history of antispasticity medication, although a tendency of increased baseline presynaptic D1 inhibition with medication might have been present (Figure S6A). We had recruited participants with clinically complete and incomplete SCI based on previous research showing alleviation of spasticity by TSCS across the severity spectrum of SCI.^9^^,^^10^ As stated earlier, we observed postsynaptic reciprocal Ia facilitation instead of inhibition^28^ in two of the three participants with SCI classified as AIS A at baseline and the strongest level of postsynaptic reciprocal Ia inhibition in the individual with an AIS-D SCI^54^ (Figure S6B). The other measures of spinal inhibition did not show any clear separation by SCI severity. The study participants in the SCI and neurologically intact groups were not matched for age, with a difference of 10 years between group means. A potential influence of age on spinal inhibitory mechanisms has been previously reported, yet, between groups of neurologically intact individuals separated by 45–55 years^94^^,^^95^ or with conflicting results on the relationship between age and spinal inhibition.^94^^,^^96^ Levels of post- and presynaptic inhibition in participants with SCI of the present study, divided according to age, do not suggest a relationship (Figure S6C). Formal statistical stratification according to antispasticity medication, SCI severity, and age would require larger sample sizes.

We here adopted the stimulation parameters used in earlier studies of EES and TSCS demonstrating alleviation of spasticity and carryover effects.^3^^,^^4^^,^^8^^,^^9^^,^^10^^,^^62^^,^^63^ It should be noted, however, that no study so far has been specifically designed to identify optimal stimulation frequency bands and amplitudes that enhance residual motor control, alleviate spasticity, and induce carryover effects.

### Conclusion

As is often the case in medicine, recent advancements in demonstrating the efficacy of spinal cord stimulation may have outpaced our scientific understanding of the underlying mechanisms. To solidify the future position of TSCS in clinical practice and instill confidence in both healthcare professionals and patients, knowledge of how this neuromodulation method interacts with spinal cord circuits is crucial. We have shown that antispasticity TSCS harnesses inhibitory mechanisms intrinsic to the spinal cord. TSCS provides activating synaptic inputs to inhibitory circuits, thereby transiently improving their function, rather than depressing overall spinal excitability in individuals who already have a diminished voluntary drive, as is the case with antispasticity medications. This distinctive mechanism may be essential for understanding how reductions in spasticity as well as improvements in residual motor control can both occur with spinal cord stimulation, be it transcutaneous or epidural. From a pathophysiological point of view, our results provide support for the long-held hypotheses that altered function of pre- and postsynaptic spinal inhibitory circuits indeed plays a causal role in spasticity following SCI in humans.

## Resource availability

### Lead contact

Requests for further information should be directed to and will be fulfilled by the lead contact, Ursula Hofstoetter (ursula.hofstoetter@meduniwien.ac.at).

### Materials availability

This study did not generate new materials or new unique reagents.

### Data and code availability

• Data reported in this paper will be shared by the lead contact upon request.
• This paper does not report the original code.
• Any additional information required to reanalyze the data reported in this paper is available from the lead contact upon request.

## Acknowledgments

The study was supported by the 10.13039/501100002428Austrian Science Fund (FWF), project no. I 3837-B34.

## Author contributions

Conceptualization, U.S.H. and K.M.; methodology, K.M., B.F., and U.S.H.; software, U.S.H.; analysis, K.M. and U.S.H.; data curation, P.L., B.F., and U.S.H.; data interpretation, K.M., U.S.H., and P.L.; writing – original draft, K.M. and U.S.H.; review & editing, all authors; visualization, U.S.H.; supervision, U.S.H., B.F., and P.L.; funding acquisition, U.S.H.

## Declaration of interests

The authors declare no competing interests.

## STAR★Methods

### Key resources table

**Table:** 

REAGENT or RESOURCE	SOURCE	IDENTIFIER
**Software and algorithms**		

MATLAB R2020a	The MathWorks, Inc.	https://www.mathworks.com
IBM SPSS Statistics 28.0.1.1	IBM Corporation	https://www.ibm.com/spss

**Other**		

Surpass system	This paper	https://www.emsbiomed.com/products/emg-ep/surpass

### Experimental model and subject details

Data were collected from ten individuals with traumatic, chronic SCI (mean age 36.9 ± 13.6 years, eight males) and twenty neurologically intact volunteers (mean age 26.8 ± 7.3 years, twelve males). Participants with SCI were recruited from the clinical program specialized in the treatment of individuals with spinal spasticity at the Neurological Center, Clinic Penzing. Inclusion criteria for the individuals with SCI were a traumatic, chronic injury (≥12 months post-onset) classified as AIS A-D^97^ with neurological levels at C3-T10, preserved reflex activity of the lumbosacral spinal cord, and spasticity in the lower limbs as a major subjective complaint. Previous studies had shown that TSCS alleviated spasticity in individuals meeting these criteria.^8^^,^^9^^,^^61^ According to the International Standards for Neurological Classification of Spinal Cord Injury,^97^ three of the participants had a motor- and sensory complete SCI classified as AIS A, six a motor and sensory incomplete SCI classified as AIS C, and one a motor and sensory incomplete SCI classified as AIS D (Table S1). The presence of spasticity affecting the lower limbs was determined at enrollment by clinically evaluating spastic hypertonia based on the MAS^77^ and rating muscle spasms using the Penn Spasms Frequency Scale.^98^ A comprehensive MAS sum score ranging from 0 (no increase in muscle tone) to 96 was calculated from individual MAS scores derived from twelve separate movements around the hip, knee, and ankle joints bilaterally.^10^^,^^53^ The MAS sum scores in the participants with SCI ranged from 12 to 59. All participants with SCI were affected by spasms, ranging from mild forms induced by stimulation to severe forms occurring more than ten times per hour.^98^ Four individuals had taken oral antispasticity medication (baclofen, elimination half-life 3–6 h; tizanidine, 1–3 h)^99^^,^^100^^,^^101^^,^^102^ either 12 or 24 h prior to participation (Table S1). Exclusion criteria included metal implants at vertebral level T10 or caudal, such as EES systems or osteosynthesis material.

The pilot study was approved by the Ethics Committee of the City of Vienna (EK 18-286-0119) and registered prior to subject enrollment (clinicaltrials.gov identifier: NCT03886857). Individuals provided written informed consent in accordance with the Declaration of Helsinki prior to their participation. Data from individuals with SCI and neurologically intact individuals were collected contemporaneously.

### Method details

#### Data acquisition

Surface-EMG was recorded bilaterally from rectus femoris (RF), biceps femoris (BF), tibialis anterior (TA), and SOL using pairs of silver-silver chloride electrodes (Intec Medizintechnik GmbH, Klagenfurt, Austria) placed with an inter-electrode distance of 3 cm according to the recommendations for Surface Electromyography for the Non-Invasive Assessment of Muscles (www.seniam.org). The ground electrode was placed over the fibular head for protocols not requiring peroneal nerve stimulation or else over the medial malleolus. Abrasive paste (Nuprep, Weaver and Company, Aurora, CO) was used for skin preparation to reduce EMG electrode resistance to less than 5 kΩ. EMG signals were recorded using the Surpass system (EMS Handels-GesmbH, Korneuburg, Austria) set to a gain of ±16 mV over a bandwidth of 15 Hz to 5 kHz and digitized at 50k samples per second and channel. All recordings were made with the participants lying in the supine position.^8^^,^^9^^,^^10^^,^^37^^,^^53^^,^^103^

#### Study protocols and stimulation procedures

We applied electrophysiological protocols to investigate post- and presynaptic spinal inhibitory mechanisms (Figures 1 and S2). The same assessments were performed in the neurologically intact individuals to establish normative data, and in the participants with SCI before (baseline evaluation E0) and twice after (evaluations E1 and E2) a 30-min session of 50-Hz TSCS applied at amplitudes below the PRM reflex threshold, see below. E1 started median 3 min (interquartile range, 0–5 min) and lasted until 75 min (70–80 min) post-TSCS. E2 started 120 min (116–128 min) and lasted until 190 min (186–195 min) post-TSCS. All protocols were performed unilaterally.

For the conditioning-test paradigms, we used the two current-controlled stimulators of the Surpass system, set to generate monophasic, rectangular stimulation pulses of 1-ms width and connected to self-adhesive hydrogel surface electrodes (Schwamedico GmbH, Ehringshausen, Germany).

The SOL-H reflex was evoked by stimulation of the tibial nerve with the cathode (Ø 3.2 cm) in the popliteal fossa and the anode (5 × 9 cm) over the anterior aspect of the knee. The cathode position was adjusted so that stimulation produced isolated plantarflexion at the ankle.

Recruitment curves of the H reflex and the M wave were obtained by increasing the stimulation amplitude in 2-mA increments from below threshold to supramaximal for the M wave (Figure S7A). Five stimuli were applied every 5 s for each stimulation amplitude. Maximum peak-to-peak amplitudes of H reflexes (H_max_) and M waves (M_max_) were determined to calculate the H_max_/M_max_ ratio.

For all subsequent conditioning-test paradigms, stimulation amplitudes were set such that unconditioned H reflexes equal to 20% M_max_ were evoked. In the SCI group, M_max_ was determined separately in E0, E1 and E2.

To assess low-frequency depression, trains of 30 stimulation pulses were applied at frequencies of 0.1, 0.2, 0.5, 1, 2, 5 and 10 Hz in a randomized order.^30^^,^^52^

To assess postsynaptic reciprocal Ia and presynaptic D1 inhibition, single conditioning stimuli were applied to the deep peroneal nerve. The cathode was placed just distal to the fibular head^44^^,^^104^^,^^105^ and the anode over the tibia, caudal to the patella (both electrodes Ø 2 cm, Spes Medica Srl, Genova, Italy). Care was taken to elicit a pure dorsiflexion without eversion of the foot.^38^^,^^58^^,^^106^ The stimulation amplitude was then set at 1.1 times the threshold that elicited a visible TA contraction.^107^ Conditioning effects on the SOL-H reflex were determined for conditioning-test intervals (CTIs) of 1, 2, 3, 4 and 5 ms for postsynaptic reciprocal Ia inhibition^35^^,^^43^ and 10, 15, 20, 25 and 30 ms for presynaptic D1 inhibition.^36^^,^^44^ Conditioning-test stimuli alternated with control stimuli (applied to evoke an H reflex without a preceding conditioning stimulus), with 5–10 s between repetitions. Ten conditioned and ten control responses were collected per CTI.

To assess ongoing presynaptic inhibition on heteronymous Ia facilitation of the SOL-H reflex, single conditioning stimuli were applied to the femoral nerve with the cathode (Ø 3.2 cm) placed over the femoral triangle and the anode (5 × 9 cm) laterally over the femoral head. The stimulation amplitude was set at four times the threshold that elicited a visible RF contraction (cf. Figure S7B).^31^ Conditioning effects on the SOL-H reflex were determined for CTIs of −9.0 to −5.6 ms, in 0.2-ms increments (negative CTIs because the conditioning stimulation site is closer to the spinal cord).^50^ Conditioning-test stimuli alternated with control stimuli, with 5–10 s between repetitions. Ten conditioned and ten control responses were collected per CTI.

In the SCI group, the protocols were performed on 2 separate days, in the following order: day 1, H_max_/M_max_ and low-frequency depression; day 2, postsynaptic reciprocal Ia inhibition, presynaptic D1 inhibition, and heteronymous Ia facilitation (Figure 1). The protocols were repeated in evaluations E0, E1 and E2. In the neurologically intact group, the protocols were conducted in the same order as in the SCI group, on 1 or 2 days, depending on individual availabilities.

In the SCI group, an EMG-based assessment of different clinical manifestations of spinal spasticity (Figure S1) was performed in all three evaluations and on both days.^10^^,^^53^ It consisted of the evaluation of tonic stretch reflexes related to hypertonia assessed by passive unilateral hip and knee flexion-extension movements (3 s each for flexion, holding the hip and knee flexed at 90°, and extension), the elicitation of cutaneous-input evoked spasms by non-noxious stimulation of the plantar surface with a blunt rod as to elicit Babinski’s sign, and the elicitation of Achilles clonus by brisk manual ankle dorsiflexion, while EMG was continuously recorded from RF, BF, TA, and SOL. All tests were repeated three times on both sides, separated by 10-s periods of no detectable EMG activity. The assessments were not performed in participants 6 and 9 and only in E0 and E1 of the first day in participant 10 due to time constraints.

#### Transcutaneous spinal cord stimulation

Lumbar TSCS was delivered through self-adhesive surface electrodes (Schwamedico GmbH), with one electrode (5 × 9 cm) placed longitudinally over the T11 and T12 spinal processes (Figure S3A) so as to overlie the spinal cord segments innervating the lower extremities.^10^^,^^53^^,^^108^ A pair of interconnected electrodes (each 8 × 13 cm) was placed on the lower abdomen, left and right of the umbilicus. A current-controlled stimulator (Stimulette r2x-S1, Dr. Schuhfried Medizintechnik GmbH, Mödling, Austria) was used to deliver charge-balanced, symmetrical, biphasic rectangular pulses of 1-ms width per phase. With reference to the abdominal electrodes, the paraspinal electrode acted as the anode for the first and as the cathode for the second phase of the pulses.^7^^,^^53^ According to our experience, such polarity results in the lowest thresholds for eliciting PRM reflexes in the lower limbs^5^ and thus for the recruitment of proprioceptive afferent fibers in the lumbar posterior roots. Thereby, the evoked responses are initiated at the abrupt change in polarity of the biphasic stimulation pulses (cf. Figure S1 in Hofstoetter et al.^7^).

The segmental stimulation site of the paraspinal electrode placed over the T11 and T12 spinal processes was tested by single pulses applied to elicit PRM reflexes bilaterally in the L2-S2 innervated RF, BF, TA and SOL muscles.^5^^,^^61^^,^^108^ Posterior root and hence proprioceptive afferent stimulation was verified by applying double pulses at interstimulus intervals of 100 ms, 50 ms, and 30 ms for assessing post-stimulation depression of the responses elicited by the second stimuli of each pair (Figure S3B).^7^^,^^10^

For the intervention, participants remained in the supine position, with additional pillows placed under their knees to avoid full leg extension, which could exacerbate spasticity.^9^^,^^53^ In lumbar TSCS, the efficacy of posterior vs. anterior root stimulation depends on the body position and spine curvature.^37^^,^^103^ The supine position secures the reliable stimulation of proprioceptive fibers within the posterior roots and consistent stimulation conditions for the period of stimulation. Antispasticity TSCS was applied at 50 Hz and amplitudes corresponding to 90% the PRM reflex threshold for 30 min. Such stimulation amplitude would recruit a proportion of the Ia afferent fibers at the subliminal fringe, without evoking lower limb muscle activity.^109^ Tonic 50-Hz stimulation of proprioceptive afferents for 30 min was previously shown to temporarily modulate spinal^8^^,^^9^^,^^61^ and supraspinal^62^^,^^63^^,^^64^ activity, with carryover effects lasting for 2 h or more. The stimulation amplitude was slowly increased to a target intensity of 90% the PRM-reflex threshold of the first muscle to respond and was subsequently applied for 30 min.^10^^,^^53^ The PRM-reflex thresholds did not differ between day 1, 35.3 ± 16.2 mA (mean ± SD), ranging from 15 to 70 mA, and day 2, 38.3 ± 13.8 mA, 14–63 mA, paired Student’s t test, t_9_ = −2.209, *p* = 0.055, r = 0.699. The stimulation amplitude for the intervention was, day 1, 31.5 ± 14.3 mA, 14–63 mA, corresponding to 90% ± 4% of the PRM-reflex threshold, and day 2, 34.6 ± 12.3 mA, 20–63 mA, 90% ± 1%. As the stimulation amplitude of the 50 Hz TSCS was increased, participants were asked whether they perceived paraesthesias (tingling sensations) in L2-S2 innervated dermatomes. Paraesthesias were reported by six of the participants, and occurred, day 1, at 27.3 ± 7.9 mA, 18–36 mA, corresponding to 72 ± 19% of the PRM-reflex threshold, and day 2, at 28.3 ± 9.7 mA, 19–45 mA, 70 ± 18% (Figure S3C). Previous studies of EES and TSCS for spasticity control had set stimulation amplitudes such that paraesthesias in lower limb dermatomes were induced without activation of lower limb muscles.^3^^,^^4^^,^^10^^,^^53^ Participants 1–3 with a sensory and motor complete SCI and participant 7 reported no paraesthesias.

### Quantification and statistical analysis

Analyses were performed using MATLAB R2020a (The MathWorks, Inc., Natick, MA, USA) and IBM SPSS Statistics 28.0.1.1 for Windows (IBM Corporation, Armonk, NY, USA) after data collection was completed for all study participants.

Assumptions of normality were tested using Shapiro-Wilk tests, and if necessary, data were transformed (ln transformation). α-errors of *p* < 0.05 (two-sided) were considered significant for all statistical tests and are reported together with the effect sizes, the partial eta-squared (${ŋ}_{p}^{2}$) for LMMs, Cohen’s f^2^ for linear regressions, or else by the correlation coefficient r. Effect sizes were considered small for 0.01 ≤ ${ŋ}_{p}^{2}$ < 0.06, 0.02 ≤ Cohen’s f^2^ < 0.15, and 0.10 ≤ r < 0.30; medium for 0.06 ≤ ${ŋ}_{p}^{2}$ < 0.14, 0.15 ≤ Cohen’s f^2^ < 0.35, and 0.30 ≤ r < 0.50, and large ${ŋ}_{p}^{2}$ ≥ 0.14, Cohen’s f^2^ ≥ 0.35, and r ≥ 0.50.^110^ All post-hoc tests were Bonferroni-corrected to correct for multiple comparisons. Descriptive statistics are reported as mean ± SE.

Care was taken to ensure consistent stimulation conditions throughout the experiments and to elicit control-H reflexes with peak-to-peak amplitudes of 20% M_max_.^47^^,^^48^ Control and the subsequently elicited conditioned H reflexes were removed from analysis when control peak-to-peak amplitudes were below 10% or above 30% M_max_.^31^^,^^48^ Peak-to-peak amplitudes of the remaining control-H reflexes normalized to M_max_ did not differ between the neurologically intact and SCI groups for the protocols assessing postsynaptic reciprocal Ia inhibition, 17.8 ± 2.3% vs. 20.4 ± 3.7%, F_1;28_ = 4.125, *p* = 0.052, $\eta_{p}^{2}$ = 0.128, heteronymous Ia facilitation, 17.6 ± 3.3% vs. 19.0 ± 3.1%, F_1;28_ = 1.186, *p* = 0.285, $\eta_{p}^{2}$ = 0.041, and low-frequency depression, 20.2 ± 2.5% vs. 26.3 ± 2.0%, F_1;28_ = 3.266, *p* = 0.082, $\eta_{p}^{2}$ = 0.104. For the protocol assessing presynaptic D1 inhibition, they were smaller in the SCI group, 17.1 ± 2.8% vs. 20.4 ± 3.6%, F_1;28_ = 6.622, *p* = 0.016, $\eta_{p}^{2}$ = 0.192.

Conditioned H reflexes were normalized to the immediately preceding controls and mean ratios were calculated for each CTI and participant, and group means ± SE were obtained.

Maximum postsynaptic reciprocal Ia inhibition was identified as the minimum conditioned-to-control response size ratio at a CTI of 2 ms or 3 ms.^43^^,^^49^^,^^58^^,^^59^ In the SCI group, the same CTI as identified in E0 was used in E1 and E2. Maximum presynaptic D1 inhibition was identified at a CTI of 15–25 ms.^29^^,^^60^

The onset of heteronymous Ia facilitation, i.e., the first CTI with the conditioned SOL-H reflex exceeding the control reflex by at least 5%,^31^^,^^45^^,^^50^ was observed at −7.6 ± 0.6 ms across participants. The CTI selected to determine heteronymous Ia facilitation was −7.2 ± 0.6 ms, i.e., 0.4 ms after the facilitation onset to obtain sizable, yet uncontaminated monosynaptic facilitation.^31^^,^^45^ Later data points, and thus the time courses of facilitation, were not considered for further analysis as they are contaminated by non-monosynaptic sources.^45^

To test whether a 30-min session of antispasticity TSCS would transiently improve the H_max_/M_max_ ratio in the individuals with SCI, separate paired Student’s t-tests were run to compare E0 to E1 and E0 to E2. To test whether TSCS would improve post- and presynaptic inhibition in the SCI group, a GLMM with evaluation (E0, E1) and outcome measure (maximum postsynaptic reciprocal Ia inhibition, maximum presynaptic D1 inhibition, heteronymous Ia facilitation) as fixed factors and subject as random factor was fitted. A separate GLMM was run to investigate spinal inhibitory circuits’ functions in E2 compared to baseline. Linear regression models were used to test for significant relationships between outcome measures. E0, E1, and E2 levels of the SCI group were separately compared to the normative levels of the neurologically intact group by GLMMs with subject group and outcome measure as fixed factors and subject as random factor.

To assess low-frequency depression, the peak-to-peak amplitudes of the 11^th^-30^th^ H reflexes elicited at each stimulation frequency were calculated. The respective mean values were normalized to the mean peak-to-peak amplitude of the 30 H reflexes at 0.1 Hz. The resulting low-frequency depression curve of E0 in the SCI group was compared to that of E1 and E2, respectively, by GLMMs. The E0, E1, and E2 low-frequency depression curves were compared to that of the neurologically intact group by fitting separate GLMMs.

For the EMG-based assessment of spasticity, the sum of the EMG-root mean square (RMS) values across muscles of the manipulated lower limb were determined.^10^^,^^53^ The time window of the calculation was from movement onset to offset for the passive hip and knee flexion-extension movements, and 5 s from the onset of manipulation for cutaneous input-evoked spasms and Achilles clonus.^10^^,^^53^ Achilles-clonus duration was measured from the onset of manipulation to the last detectable bout of EMG activity. Mean values were obtained by averaging over the three repetitions. Results in E1 and E2 were considered improved if mean EMG-RMS values or Achilles-clonus durations were below baseline. The results obtained in E0 were compared to those in E1 and E2, respectively, using GLMMs with evaluation and spasticity measure as fixed factors and subject as random factor.

#### Additional resources

This study was registered prior to subject enrollment (clinicaltrials.gov identifier: NCT03886857).

## Supplemental information

Document S1. Figures S1–S7 and Tables S1–S5

Document S2. Article plus supplemental information
