# Supplementary material for: Transcutaneous spinal cord stimulation neuromodulates pre- and postsynaptic inhibition in the control of spinal spasticity
Source: Cell Rep Med. 2024 Nov 11;5(11):101805. doi: 10.1016/j.xcrm.2024.101805 (PMC11604492; doi:10.1016/j.xcrm.2024.101805)
Supplement: Document S1. Figures S1–S7 and Tables S1–S5 [file mmc1.pdf]

Cell Reports Medicine, Volume *5*

Supplemental information

Transcutaneous spinal cord stimulation neuromodulates pre- and postsynaptic inhibition in the control of spinal spasticity

Karen Minassian, Brigitta Freundl, Peter Lackner, and Ursula S. Hofstoetter

Table S1. Neurological status of study participants with spinal cord injury. Related to Figure 1.

Subj. nr.	Sex	Age (y)	Neurol. level of SCI	Years post-SCI	AIS grade	LEMS total (max. 50)	PP sensory subscore, L1–S2 (max. 28)	LT sensory subscore, L1–S2 (max. 28)	LE MAS sum score (max. 96)	Penn spasm frequency score (max. 4)	Penn spasm severity score (max. 3)	Antispasticity medication (daily dosage)
1	M	61	C5	7	A	0	0	0	39	2	3	None
2	M	32	T7	3	A	0	0	0	22	2	2	None
3	M	34	T5	2	A	0	0	0	12	4	2	25 mg baclofen
4	M	48	C2	31	C	0	0	14	14.5	1	2	45 mg baclofen
5	F	28	T3	6	C	2	0	3	55.5	2	3	None
6	F	19	T7	1	C	2	3	3	39.5	3	3	75 mg baclofen, 10 mg tizanidine
7	M	31	C3	11	C	4	14	14	54	3	2	50 mg baclofen
8	M	50	T3	31	C	5	4	14	24	2	2	None
9	M	45	C5	29	C	14	14	14	59	3	2	None
10	M	21	T4	2	D	22	0	13	19	2	2	None

AIS, American Spinal Injury Association Impairment Scale; LE, lower extremity; LEMS, lower extremity motor score; LT, light touch; MAS, Modified Ashworth Scale; neurol., neurological; nr., number; PP, pin prick; SCI, spinal cord injury; subj., subject; y, years.

Table S2. Monosynaptic reflex excitability. Related to Figure 1.

	**Mean H_{max}/M_{max} ratio ± SD**
**SCI group, evaluation E0**	0.51 ± 0.08
**SCI group, evaluation E1**	0.45 ± 0.09
**SCI group, evaluation E2**	0.50 ± 0.09
**Neurologically intact group**	0.52 ± 0.05
**Statistical analyses: Results of paired Student's t-tests**	
SCI group, evaluation E0 vs. E1	t_9 = 2.756, p = .022, r = .922††††
SCI group, evaluation E0 vs. E2	t_9 = 0.560, p = .589, r = .187
**Statistical analyses: Results of generalized linear mixed models**	
SCI group (E0) vs. neurologically intact group	$F_{1;28}$ = 0.009, p = .927, η_p^2 = 0.0003
SCI group (E1) vs. neurologically intact group	$F_{1;28}$ = 0.472, p = .498, η_p^2 = 0.017
SCI group (E2) vs. neurologically intact group	$F_{1;28}$ = 0.051, p = .823, η_p^2 = 0.002

For comparisons of monosynaptic reflex excitability within the SCI group, paired Student's t-tests were performed, and for between-group comparisons, generalized linear mixed models with subject group and stimulation frequency as fixed factors and subject as random factor were run. SCI, spinal cord injury; E0, baseline evaluation before a 30-minute session of antispasticity transcutaneous spinal cord stimulation (TSCS); E1, E2, post-TSCS evaluations; ††††, large effect size.

Table S3. Maximum levels of post- and presynaptic inhibition. Related to Figure 2.

	Group means ± SE		
	**Postsynaptic reciprocal Ia inhibition**	**Presynaptic D1 inhibition**	**Heteronymous Ia facilitation**
**SCI group, evaluation E0**	0.920 ± 0.054	0.907 ± 0.063	1.357 ± 0.082
**SCI group, evaluation E1**	0.802 ± 0.028	0.774 ± 0.071	1.266 ± 0.078
**SCI group, evaluation E2**	0.943 ± 0.033	0.839 ± 0.063	1.255 ± 0.090
**Neurologically intact group**	0.807 ± 0.021	0.617 ± 0.043	1.182 ± 0.082

SCI, spinal cord injury; E0, baseline evaluation before a 30-minute session of antispasticity transcutaneous spinal cord stimulation (TSCS); E1, E2, post-TSCS evaluations.

Table S4. Low-frequency depression of soleus-H reflexes. Related to Figure 4.

	Test frequency						
	**0.1 Hz**	**0.2 Hz**	**0.5 Hz**	**1.0 Hz**	**2.0 Hz**	**5.0 Hz**	**10.0 Hz**
**SCI group, evaluation E0**	0.994 ± 0.009	0.829 ± 0.048	0.608 ± 0.070	0.534 ± 0.062	0.741 ± 0.223	0.691 ± 0.152	0.869 ± 0.252
**SCI group, evaluation E1**	1.005 ± 0.147	0.836 ± 0.082	0.644 ± 0.080	0.586 ± 0.094	0.636 ± 0.131	0.682 ± 0.112	0.807 ± 0.147
**SCI group, evaluation E2**	1.022 ± 0.024	0.889 ± 0.067	0.697 ± 0.068	0.610 ± 0.103	0.599 ± 0.123	0.565 ± 0.092	0.782 ± 0.149
**Neurologically intact group**	1.013 ± 0.006	0.779 ± 0.036	0.467 ± 0.030	0.282 ± 0.039	0.186 ± 0.025	0.127 ± 0.023	0.206 ± 0.052

Statistical analyses: Results of generalized linear mixed models

**SCI group, evaluation E0 vs. E1**	
Factor evaluation (E0, E1)	$F_{1;126} = 0.031$, p = .861, $\eta_p^2 = 0.0002$
Evaluation x frequency interaction	$F_{6;126} = 0.140$, p = .991, $\eta_p^2 = 0.007$
**SCI group, evaluation E0 vs. E2**	
Factor evaluation (E0, E2)	$F_{1;126} = 0.061$, p = .796, $\eta_p^2 = 0.0005$
Evaluation x frequency interaction	$F_{6;126} = 0.455$, p = .840, $\eta_p^2 = 0.021$
**SCI group (E0) vs. neurologically intact group**	
Factor subject group	$F_{1;196} = 22.927$, p < .001, $\eta_p^2 = 0.154^{††††}$
Subject group x frequency interaction	$F_{6;196} = 8.111$, p < .001, $\eta_p^2 = 0.279^{††††}$
**SCI group (E1) vs. neurologically intact group**	
Factor subject group	$F_{1;196} = 32.492$, p < .001, $\eta_p^2 = 0.205^{††††}$
Subject group x frequency interaction	$F_{6;196} = 11.237$, p < .001, $\eta_p^2 = 0.349^{††††}$
**SCI group (E2) vs. neurologically intact group**	
Factor subject group	$F_{1;196} = 52.824$, p < .001, $\eta_p^2 = 0.295^{††††}$
Subject group x frequency interaction	$F_{6;196} = 8.339$, p < .001, $\eta_p^2 = 0.284^{††††}$

Values are mean peak-to-peak amplitudes (± SE) of the 11th-30th soleus-H reflexes elicited at repetition rates as indicated (test frequency), normalized to the 1st-30th H reflexes at 0.1 Hz. For comparisons of low-frequency depression within the SCI group, evaluation and stimulation frequency served as fixed factors of the generalized linear mixed models, and for between-group comparisons, subject group and stimulation frequency; subject was included as random factor in all models. SCI, spinal cord injury; E0, baseline evaluation before a 30-minute session of antispasticity transcutaneous spinal cord stimulation (TSCS); E1, E2, post-TSCS evaluations; ††††, large effect size.

Table S5. Electromyography-based assessment of clinical manifestations of spasticity. Related to Figure 5.

	Tonic stretch reflexes, EMG-RMS (μV)	Cutaneous input-evoked spasms, EMG-RMS (μV)	Achilles clonus, EMG-RMS (μV)	Achilles clonus, duration (s)
**SCI group, evaluation E0**	41.0 ± 6.1	39.0 ± 6.6	48.7 ± 5.9	8.6 ± 2.0
**SCI group, evaluation E1**	26.8 ± 4.2	24.8 ± 4.3	29.5 ± 3.7	5.4 ± 1.5
**SCI group, evaluation E2**	14.3 ± 4.2	26.5 ± 7.9	28.9 ± 5.3	2.0 ± 0.5

EMG, electromyography; RMS, root-mean-square; SCI, spinal cord injury; E0, baseline evaluation before a 30-minute session of antispasticity transcutaneous spinal cord stimulation (TSCS); E1, E2, post-TSCS evaluations.

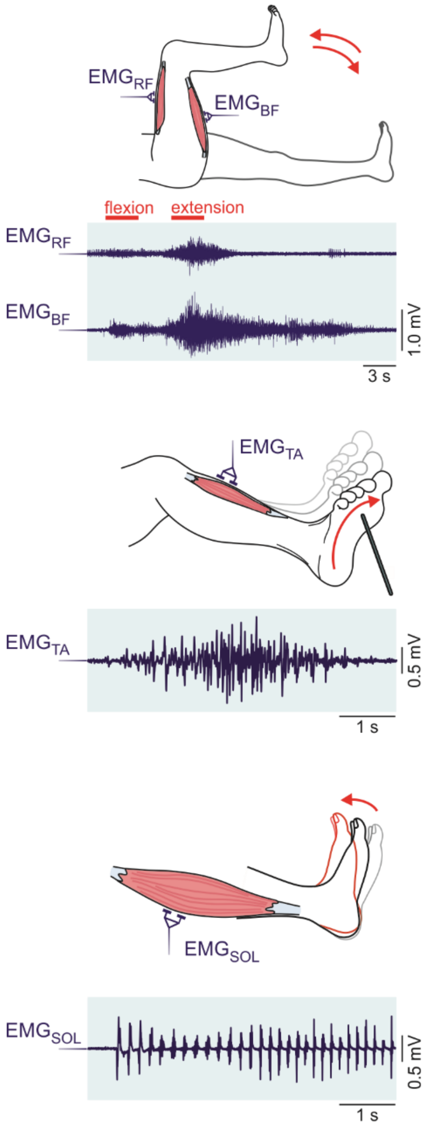

(A) Tonic stretch reflexes

While tendon tap reflexes can be evoked in neurologically intact individuals, slow muscle stretch for a few seconds does not produce any reflex contraction. In individuals with spinal spasticity, on the contrary, such tonic stretch reflexes can also be elicited. Here, we assessed tonic stretch reflexes by passive unilateral hip and knee flexion-extension movements, with 3 s from the starting position in extension to flexion until a 90° angle at hip and knee was reached, holding this position for 3 s, and extending the leg back for a final 3 s.

(B) Cutaneous input-evoked spasms

Muscle spasms are involuntary muscle contractions that can occur either spontaneously or in response to joint movement or skin stimulation in individuals with spinal spasticity. They can persist for several seconds even after the initial trigger has ceased. Mechanisms underlying muscle spasms include plastic changes in spinal motoneurons as a result of SCI, leading to their sustained depolarization (plateau potentials) after brief stimuli and the occurrence of self-sustained firing. Here, we assessed spasms by stroking the foot sole with a blunt rod.

(C) Achilles clonus

Clonus refers to a sequence of brief muscle contractions with a frequency of 5-8 Hz caused by a sudden stretch. It can be triggered, for instance, when transferring to a wheelchair or driving over an uneven surface and can also interfere with residual walking ability. According to the prevailing theory, it is a self-perpetuating reactivation of the stretch reflex pathway that becomes hyperactive after SCI. Here, we applied a sudden dorsiflexion to the foot to assess Achilles clonus, which manifests as an involuntary rhythmic movement around the ankle, typically lasting for a few seconds.

Figure S1. Spinal spasticity and its clinical manifestations. Related to Figure 1.

Spasticity is a common neurological impairment emerging after spinal cord injury (SCI). It debilitates motor function, contributes to the development of contractures and pain, and limits activities of daily living. Managing spasticity is hence crucial in the clinical care of patients with SCI and is also paramount to a successful rehabilitation process. The velocity-dependent increase in the tonic stretch reflex of a muscle when it is passively lengthened, experienced as an increased muscle tone, is classically considered a defining feature of spinal spasticity. More recent definitions include further signs and symptoms, such as muscle spasms and clonus.[1,2] To obtain a comprehensive picture of spasticity and document the antispasticity effects of transcutaneous spinal cord stimulation, we here assessed (**A**) tonic stretch reflexes; (**B**) cutaneous-input evoked spasms; and (**C**) Achilles clonus. BF, biceps femoris; EMG, electromyography; RF, rectus femoris; SOL, soleus; TA, tibialis anterior.

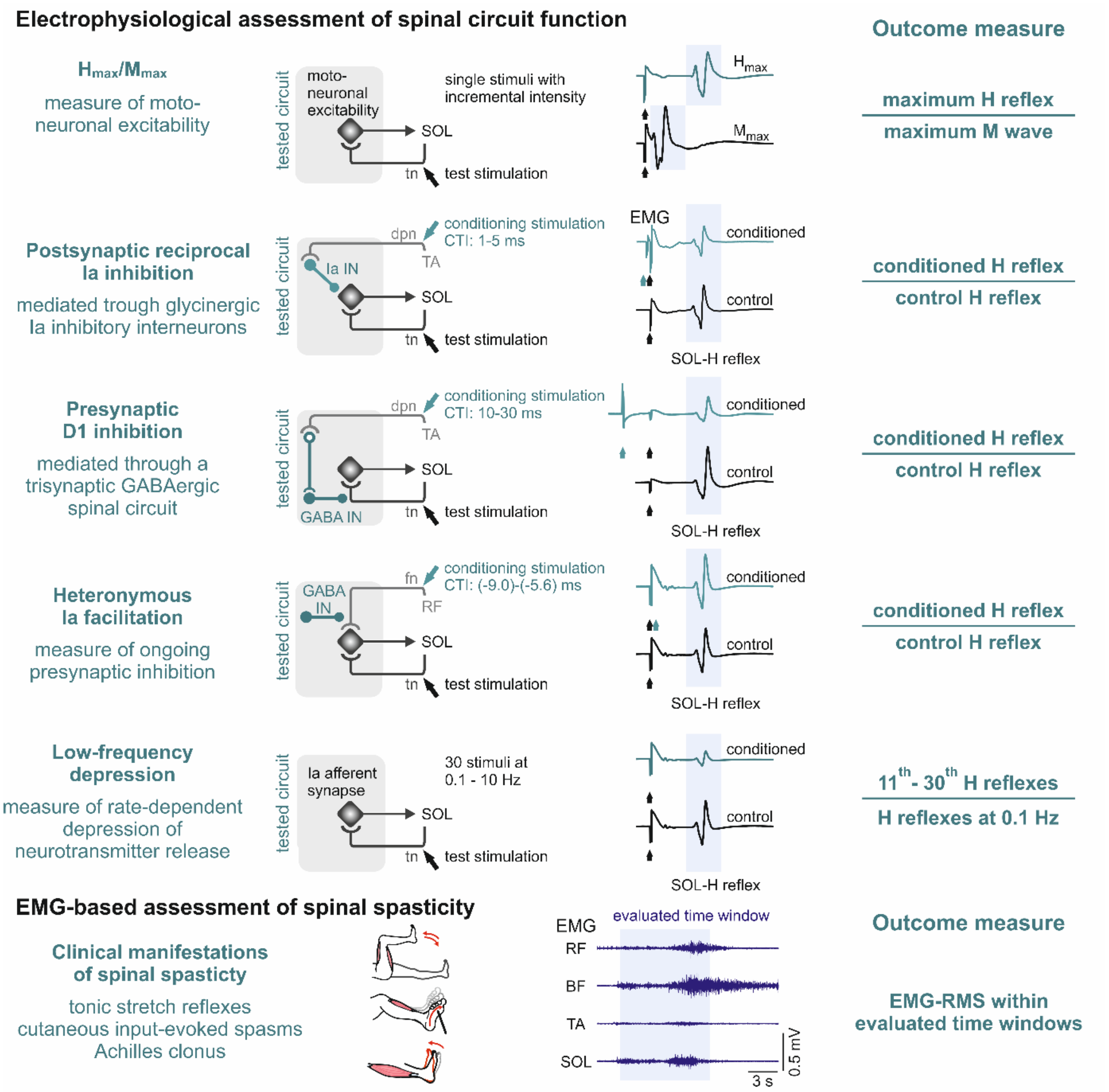

Figure S2. Overview of assessments and outcome measures. Related to Figure 1.

Motoneuronal excitability within the monosynaptic reflex circuit was estimated by the ratio of the maximum soleus (SOL)-H reflex (H_{max}) to the maximum M wave (M_{max}) peak-to-peak (P2P) amplitudes. The outcome measures that describe post- and presynaptic inhibition were derived from conditioning-test paradigms using the SOL-H reflex as a test reflex and were calculated as the P2P amplitudes of the conditioned normalized to the control H reflexes. For evaluating low-frequency depression, trains of 30 H reflexes were elicited at frequencies of 0.1-10 Hz. The peak-to-peak amplitudes of the 11th-30th H reflexes elicited at each stimulation frequency were calculated, and the respective mean values were normalized to the mean P2P amplitude of the 30 H reflexes at 0.1 Hz. Clinical manifestations of spasticity were assessed based on the root mean square (RMS) values of electromyographic (EMG) activity associated with tonic stretch reflexes, cutaneous input-evoked spasms, and Achilles clonus. BF, biceps femoris; CTI, conditioning-test interval; dpn, deep peroneal nerve; fm, femoral nerve; GABA IN, GABAergic interneuron; Ia IN, Ia inhibitory interneuron; RF, rectus femoris; TA, tibialis anterior; tn, tibial nerve.

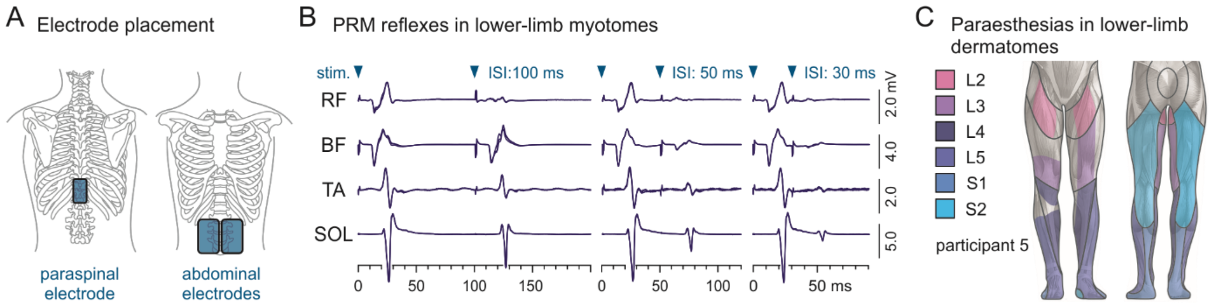

Figure S3. Transcutaneous spinal cord stimulation. Related to Figure 1.

(**A**) Transcutaneous spinal cord stimulation (TSCS) was applied through a self-adhesive electrode placed over the T11 and T12 spinous processes and a pair of interconnected electrodes over the lower abdomen.

(**B**) Stimulation of lumbosacral posterior roots was tested by applying double stimuli (stim.) at decreasing interstimulus intervals (ISI) of 100 ms, 50 ms, and 30 ms. The first stimulation pulse of each pair elicited responses in rectus femoris (RF), biceps femoris (BF), tibialis anterior (TA) and soleus (SOL) bilaterally. Post-stimulation depression of the responses to the second stimulation pulses of each pair identified these responses as posterior root-muscle (PRM) reflexes, hence confirming multisegmental proprioceptive afferent stimulation.[3–5]

(**C**) For the intervention, TSCS was applied at an intensity corresponding to 90% of the PRM reflex threshold and at 50 Hz, during which paraesthesias in L2-S2 innervated lower-limb dermatomes were produced in participants with residual sensory function. These tingling sensations complementarily confirmed the stimulation of somatosensory afferents throughout the duration of the intervention.[6,7] Exemplary distribution of paraesthesias (shaded areas) as perceived by participant 5.

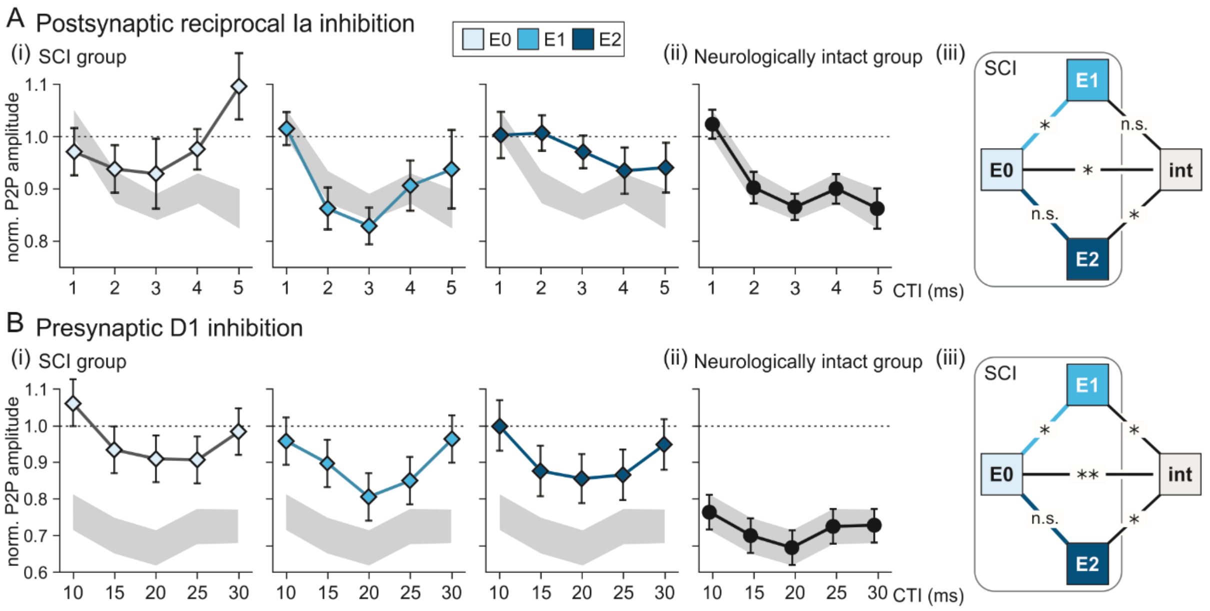

Figure S4. Time courses of post- and presynaptic inhibition. Related to Figures 2 and 3.

(**A**) Time courses of postsynaptic reciprocal Ia inhibition with condition-test intervals (CTIs) of 1-5 ms for (i) the SCI group for evaluations E0, E1, and E2 as indicated and (ii) the neurologically intact group. Diamonds and circles are means of peak-to-peak (P2P) amplitudes of conditioned reflexes normalized (norm.) to control reflexes ± SE. Grey backgrounds in (i) show the one-SE band of postsynaptic Ia inhibition of the neurologically intact group. (iii) The time course of inhibition in E0 in the SCI group differed from E1, but not from E2. The time course in the neurologically intact group (int) differed from E0 and E2 in the SCI group, but not from E1. Specifically, within the SCI group, separate generalized linear mixed models (GLMM) with evaluation (E0, E1 and E0, E2, respectively) and CTI as fixed factors and subject as random factor were run. For the comparison between E0 and E1, the factor evaluation was significant, $F_{1;81} = 6.793$, $p = .011$, $\eta_p^2 = 0.077$ (medium effect size), while the evaluation x CTI interaction was not significant, $F_{4;81} = 1.437$, $p = .229$, $\eta_p^2 = 0.066$. For the comparison between E0 and E2, neither the factor evaluation, $F_{1;\,78.111} = 0.413$, $p = .522$, $\eta_p^2 = 0.005$, nor the evaluation x CTI interaction, $F_{4;76.995} = 2.418$, $p = .056$, $\eta_p^2 = 0.112$, were significant. For the between-groups comparisons, separate GLMMs with subject group (neurologically intact, SCI) and CTI as fixed factors and subject as random factor were run for E0, E1, and E2. For E0, both the factor subject group, $F_{1;28} = 4.534$, $p = .042$, $\eta_p^2 = 0.139$ (medium effect size), and the subject group x CTI interaction, $F_{4;112} = 4.161$, $p = .004$, $\eta_p^2 = 0.129$ (medium effect size), were significant. For E1, neither the factor subject group, $F_{1;28} < 0.001$, $p = .990$, $\eta_p^2 < 0.001$, nor the subject group x CTI interaction, $F_{4;112} = 0.883$, $p = .476$, $\eta_p^2 = 0.031$, were significant. For E2, the factor subject group was significant, $F_{1;\,27.084} = 5.743$, $p = .014$, $\eta_p^2 = 0.175$ (large effect size), while the subject group x CTI interaction $F_{4;108.439} = 1.235$, $p = .300$, $\eta_p^2 = 0.044$, was not. TSCS had thus temporarily improved the time course of postsynaptic reciprocal Ia inhibition in the SCI group in evaluation E1 to normative data.

(**B**) Time courses of presynaptic D1 inhibition with CTIs of 10-30 ms for (i) the SCI group for evaluations E0, E1, and E2 as indicated and (ii) the neurologically intact group. Grey backgrounds in (i) show the one-SE band of presynaptic D1 inhibition in the neurologically intact group. (iii) The time course of inhibition in E0 in the SCI group differed from E1, but not from E2. The time course in the neurologically intact group differed from those in all three evaluations in the SCI group. Specifically, within the SCI group, a GLMM run to compare E0 and E1 revealed the factor evaluation as significant, $F_{1;81} = 6.007$, $p = .016$, $\eta_p^2 = 0.069$ (medium effect size), while the evaluation x CTI interaction was not significant, $F_{4;81} = 0.411$, $p = .800$, $\eta_p^2 = 0.020$. For the comparison between E0 and E2, neither the factor evaluation, $F_{1;77.306} = 3.775$, $p = .056$, $\eta_p^2 = 0.047$, nor the evaluation x CTI interaction, $F_{4;77.064} = 0.044$, $p = .996$, $\eta_p^2 = 0.002$, were significant. For the between-groups comparisons, separate GLMMs showed that the factor subject group was significant for all three evaluations in

the SCI group, E0, $F_{1;28} = 13.654$, $p < .001$, $\eta_p^2 = 0.328$ (large effect size), E1, $F_{1;28} = 6.998$, $p = .013$, $\eta_p^2 = 0.200$ (large effect size); and E2, $F_{1;28} = 7.104$, $p = .013$, $\eta_p^2 = 0.199$ (large effect size). The subject group x CTI interactions were not significant, E0, $F_{4;112} = 0.759$, $p = .554$, $\eta_p^2 = 0.026$; E1, $F_{4;112} = 0.920$, $p = .455$, $\eta_p^2 = 0.032$; and E2, $F_{4;109.076} = 0.610$, $p = .656$, $\eta_p^2 = 0.022$. Thus, the time courses found in the SCI group were similar in shape to that found in the neurologically intact group, but were shifted to higher values. E0, pre-TSCS evaluation; E1, E2, post-TSCS evaluations; n.s., not significant; SCI, spinal cord injury; TSCS, transcutaneous spinal cord stimulation; *, $p < .05$; **, $p < .001$.

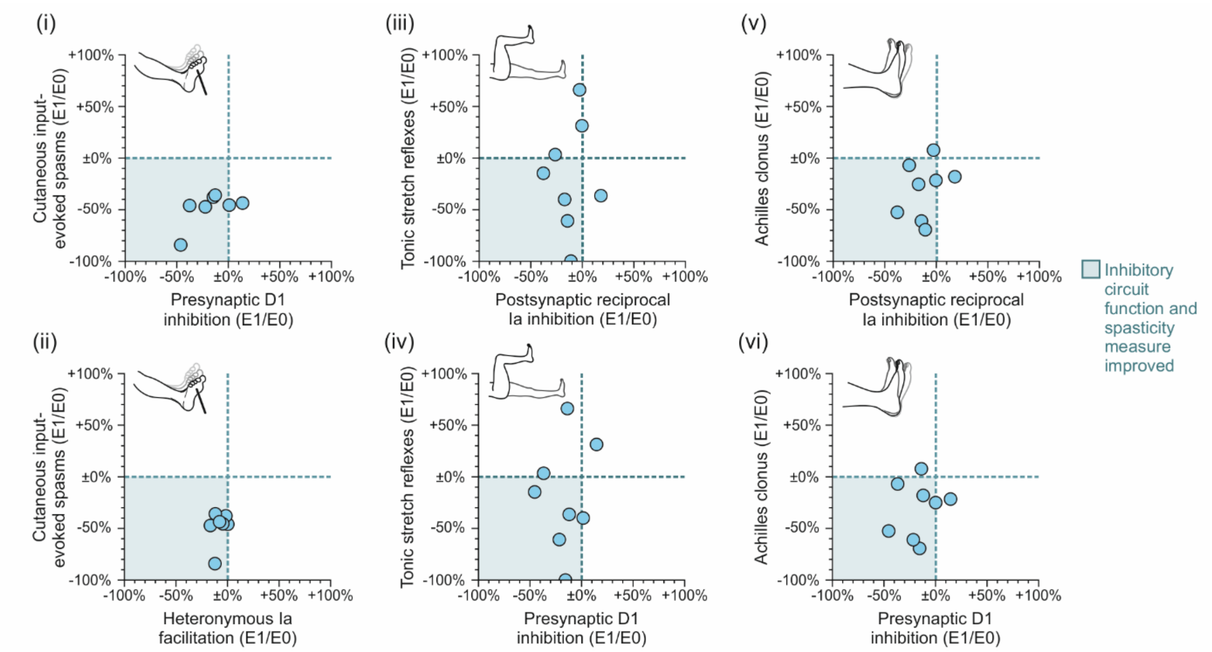

Figure S5. Summary of non-significant correlations between stimulation-induced changes in spasticity measures and changes in post- and presynaptic inhibition. Related to Figure 5.

Scatter plots show relationships between relative changes from E0 to E1 in cutaneous input-evoked spasms and (i) presynaptic D1 inhibition, r = 0.625, p = .133; and (ii) heteronymous Ia facilitation, r = 0.320, p = .485; between tonic stretch reflexes and (iii) postsynaptic reciprocal Ia inhibition, r = 0.095, p = .843; and (iv) presynaptic D1 inhibition, r = 0.125, p = .769; and between Achilles clonus and (v) postsynaptic reciprocal Ia inhibition, r = 0.326, p = .430; and (vi) presynaptic D1 inhibition, r = 0.219, p = .603. E0, pre-TSCS evaluation; E1, post-TSCS evaluation; TSCS, transcutaneous spinal cord stimulation.

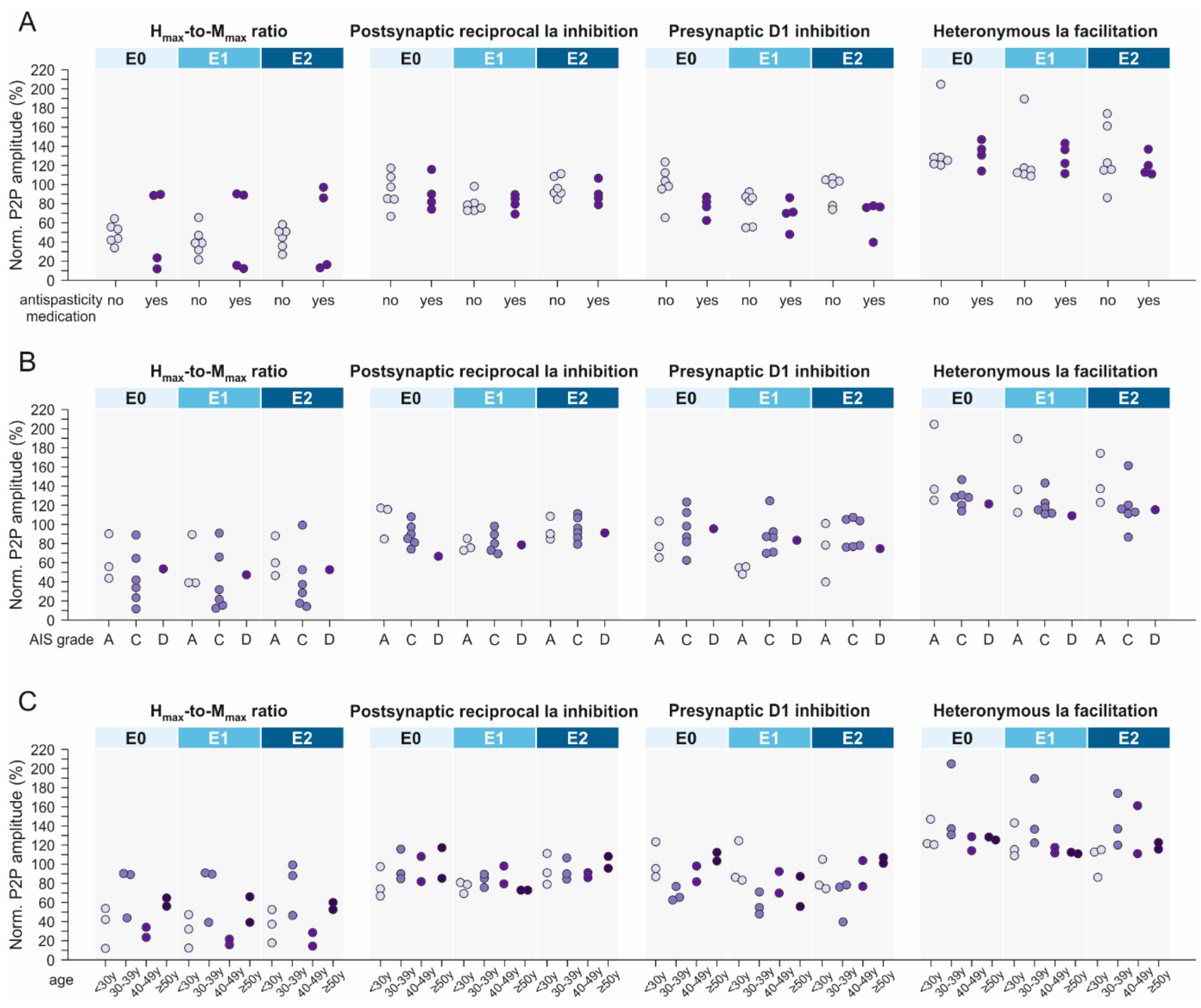

Figure S6. Measures of spinal inhibition in the spinal cord injury group separated according to (A) antispasticity medication, (B) severity of SCI, and (C) age. Related to Figure 2.

Details on the neurological status of the study participants and the use of antispasticity medication are provided in Table S1. AIS, American Spinal Injury Association Impairment Scale;[8] E0, pre-TSCS evaluation; E1, E2, post-TSCS evaluations; norm. P2P amplitude, peak-to-peak amplitude of conditioned normalized to control reflexes; TSCS, transcutaneous spinal cord stimulation.

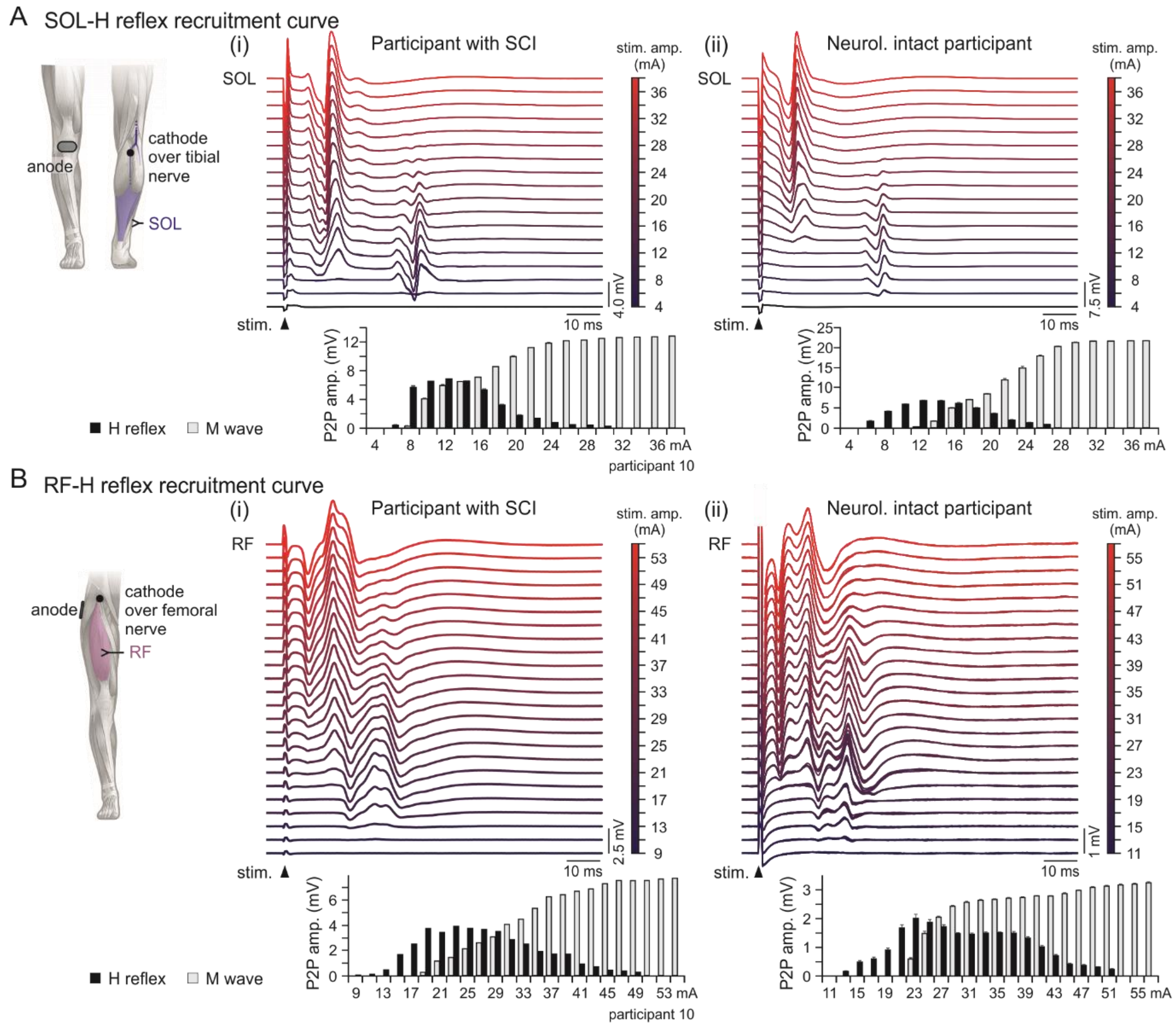

Figure S7. H reflexes and M waves in leg and thigh muscles. Related to STAR Methods.

(**A**) Electromyographic recordings of soleus (SOL) responses evoked by stimulation (stim.) of the tibial nerve, with the cathode placed in the popliteal fossa and the anode over the anterior aspect of the knee. Shown are five superimposed responses elicited with each incremental stimulation amplitude (stim. amp.) as indicated in (i) participant 10 with spinal cord injury (SCI) and (ii) a neurologically (neurol.) intact participant. Recruitment curves are illustrated by bar diagrams showing the mean peak-to-peak amplitudes (P2P amp.) + SD of the H reflexes (black) and M waves (light grey).

(**B**) Responses in rectus femoris (RF) evoked by stimulation of the femoral nerve, with the cathode placed over the femoral triangle and the anode laterally over the femoral head in (i) participant 10 with SCI and (ii) a neurologically intact participant.

Supplemental References

1. Trompetto, C., Marinelli, L., Mori, L., Pelosin, E., Currà, A., Molfetta, L., and Abbruzzese, G. (2014). Pathophysiology of Spasticity: Implications for Neurorehabilitation. Biomed Res. Int. *2014*, 1–8. 10.1155/2014/354906.

2. Pandyan, A., Hermens, H., Conway, B., and Johnson, G. (2018). Definition and Measurement of Spasticity and Contracture. In Neurological Rehabilitation. Spasticity and Contractures in Clinical Practice and Research, A. Pandyan, H. Hermens, and B. Conway, eds. (Imprint CRC Press), pp. 1–23.

3. Minassian, K., Persy, I., Rattay, F., Dimitrijevic, M.R., Hofer, C., and Kern, H. (2007). Posterior root-muscle reflexes elicited by transcutaneous stimulation of the human lumbosacral cord. Muscle Nerve *35*, 327–336. 10.1002/mus.20700.

4. Hofstoetter, U.S., Freundl, B., Binder, H., and Minassian, K. (2018). Common neural structures activated by epidural and transcutaneous lumbar spinal cord stimulation: Elicitation of posterior root-muscle reflexes. PLoS One *13*, e0192013. 10.1371/journal.pone.0192013.

5. Minassian, K., Freundl, B., and Hofstoetter, U.S. (2020). The posterior root-muscle reflex. In Neurophysiology in Neurosurgery, V. Deletis, J. Shils, F. Sala, and K. Seidel, eds. (Elsevier), pp. 239–253. 10.1016/B978-0-12-815000-9.00018-6.

6. Hofstoetter, U.S., Freundl, B., Danner, S.M., Krenn, M.J., Mayr, W., Binder, H., and Minassian, K. (2020). Transcutaneous Spinal Cord Stimulation Induces Temporary Attenuation of Spasticity in Individuals with Spinal Cord Injury. J. Neurotrauma *37*, 481–493. 10.1089/neu.2019.6588.

7. Hofstoetter, U.S., Freundl, B., Lackner, P., and Binder, H. (2021). Transcutaneous Spinal Cord Stimulation Enhances Walking Performance and Reduces Spasticity in Individuals with Multiple Sclerosis. Brain Sci. *11*, 472. 10.3390/brainsci11040472.

8. Rupp, R., Biering-Sørensen, F., Burns, S.P., Graves, D.E., Guest, J., Jones, L., Read, M.S., Rodriguez, G.M., Schuld, C., Tansey, K.E., et al. (2021). International Standards for Neurological Classification of Spinal Cord Injury. Top. Spinal Cord Inj. Rehabil. *27*, 1–22. 10.46292/sci2702-1.
